# Intermediate Habitat Fragmentation Buffers Droughts: How Individual Energy Dynamics Mediate Mammal Community Response to Stressors

**DOI:** 10.1111/gcb.70224

**Published:** 2025-05-14

**Authors:** Leonna Szangolies, Cara A. Gallagher, Florian Jeltsch

**Affiliations:** ^1^ Plant Ecology and Nature Conservation Potsdam University Potsdam Germany; ^2^ Ecoscience Aarhus University Roskilde Denmark; ^3^ Berlin‐Brandenburg Institute of Advanced Biodiversity Research (BBIB) Berlin Germany

**Keywords:** biodiversity, coexistence, combined global change effects, dynamic energy budget, individual metabolism, individual‐based model, multiple stressors, small mammals

## Abstract

Biodiversity is threatened by land‐use and climate change. Although these processes are known to influence species survival and diversity, predicting their combined effects on communities remains challenging. We here aim to disentangle the combined effects of drought‐induced resource shortage and habitat fragmentation on species coexistence. To understand how both fragmentation and droughts affect individual movement and physiology, and ultimately influence population and community dynamics, we use an individual‐based metabolic modelling approach to simulate a community of small mammals. Individuals forage in the landscape to ingest energy, which they then allocate to basal maintenance, digestion, locomotion, growth, reproduction, and storage. If individuals of several species are able to balance their energy intake and needs, and additionally store energy as fat reserves, they may overcome stress periods and coexist. We find that species recover best after a drought when they live in moderately fragmented landscapes compared to those with low or high fragmentation. In low fragmented landscapes, high local competition during resource shortages is problematic, while in highly fragmented landscapes, low energy balance and storage often lead to high mortality during drought. Intermediately fragmented landscapes balance these effects and show the least impact of droughts on species richness, a pattern that holds also when integrating observed drought time series from monitoring data in the model simulations. Due to the interacting negative impacts, we suggest that with ongoing global change, it is increasingly important to understand stressors simultaneously to identify measures that support species coexistence and biodiversity. Including individual energy dynamics allowed us to conflate the different global change effects through energy storage and energy allocation to different processes. Our presented community model, which integrates metabolic and behavioural reactions of individuals to different stressors and scales them to the community level, offers valuable insights with great potential to support nature conservation.

## Graphical abstract

Simulations of our novel individual‐based metabolic community model for a terrestrial mammal community revealed a positive effect of intermediate habitat fragmentation on coexistence when simulating habitat fragmentation and droughts as multiple stressors. Landscapes with intermediate habitat fragmentation buffered drought effects best compared to low or high fragmentation, both when simulating experimental droughts of varying length and magnitude, and when integrating observed drought timeseries from monitoring data. The energetic model allowed to conflate these different global change effects and provided insights into coexistence mechanisms with similarity in energy balance among species promoting coexistence and thus diversity.

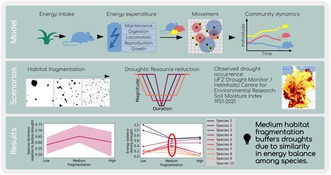

## 1 Introduction

Biodiversity is increasingly threatened by the combined impacts of global change (Garcia et al. 2014). While the effects of individual stressors, such as habitat fragmentation or extreme weather events, are frequently investigated in isolation, the interactions between multiple stressors remain less well studied and understood (Schulte to Bühne et al. 2021; Van Moorsel et al. 2023). However, in reality, global change effects rarely occur in isolation. Species are more likely to face a combination of global change effects including land‐use changes and climate changes that can have additive and interacting impacts (Berlinches de Gea et al. 2023; Jeltsch et al. 2011; Moe et al. 2013; Pirotta et al. 2022). The multitude of stressors and effects these stressors have on different biological and ecological processes, such as reproduction and movement, complicates efforts to measure and understand their combined impact on individuals and populations (Orr et al. 2020).

Additionally, populations typically do not occur in isolation, but in communities, where intricate interactions between species influence their response to environmental change (Turschwell et al. 2022; Vandvik et al. 2020; Siepielski et al. 2018). This complexity further complicates efforts to understand the impacts of global change on biodiversity, as mechanisms governing species coexistence are already challenging to untangle without changing environments. Nonetheless, understanding and predicting the impacts of global change effects on communities and biodiversity is crucial for the development of effective conservation strategies. To take a step towards this, we here aim to disentangle the impact of exemplary combined global change effects on species dynamics and coexistence of a small mammal community.

Among the various global change stressors, habitat fragmentation and droughts stand out as particularly contentious. The effects of habitat fragmentation per se are debated in ecology since decades (Debinski and Holt 2000; Fahrig et al. 2019; Fletcher Jr, Didham, et al. 2018). While habitat fragmentation is often perceived as negative for species due to, for example, patch isolation and dispersal limitations (Debinski and Holt 2000; Fletcher Jr, Reichert, et al. 2018), the benefits of smaller habitat patches, for example, by increasing landscape heterogeneity, have been repeatedly demonstrated (Lindenmayer 2019; Riva and Fahrig 2022; Szangolies et al. 2022). Previous studies suggested that a moderate level of habitat fragmentation may help equalise competition and energy balance among species (Szangolies, Gallagher, et al. 2024; Szangolies et al. 2022). However, this balance could be upset by fluctuations in resource availability that may result from climate change‐induced extreme events, such as droughts. This is particularly relevant given the global increase in drought frequency and severity (Vicente‐Serrano et al. 2022), which is projected to continue (Spinoni et al. 2018). Drought events currently threaten biodiversity (Weiss et al. 2024) and their negative effects may even have been so far underestimated (Smith et al. 2024). Despite the importance of understanding the combined impact of droughts and habitat fragmentation, research in this area remains limited, often focusing on individual species and using population persistence as the primary response variable (Hung et al. 2021; Oliver et al. 2015; Forner et al. 2020).

However, rather than causing direct mortality, these stressors often affect species indirectly by altering resource availability and consequently individual body condition or internal state. Since such changes are reflected in the energetic state of individuals, integrating energy dynamics into the study of multiple global change effects may further improve our mechanistic understanding of their combined impacts. Energy is the basis for all processes of life at the individual level (Brown et al. 2004; Tomlinson et al. 2014). Animals acquire energy through foraging to cover their energetic demands and build reserves for future needs. Besides maintenance and survival, they need energy to locate resources and move, and to invest in growth and reproduction (Brown et al. 2004). Since available energy is typically limited, trade‐offs between processes arise, for example, investing energy in movement to new locations may result in less energy available for reproduction (Pontzer and McGrosky 2022). Hence, energy dynamics are sensitive to landscape changes, such as habitat fragmentation, because individuals adapt their movement and potentially expend more energy for locomotion (Fahrig 2007; Szangolies, Gallagher, et al. 2024). Additionally, during extreme events, stored energy reserves may help individuals to survive periods of stress (Olsen et al. 2021). Furthermore, it has been suggested that energy dynamics not only mediate individual fitness but also affect coexistence within communities (Brandl et al. 2023; Pigot et al. 2016; Szangolies, Gallagher, et al. 2024).

To study animal communities from an energetic perspective, simulation models are a valuable tool that have been employed to predict species responses to disturbance (Gallagher et al. 2021; Houston et al. 2012; Pirotta et al. 2021). In contrast to currently advancing field technologies such as accelerometers, simulation models allow for tracking energy allocation to different physiological processes, studying individuals over an entire lifetime, and investigating all individuals within a community (Malishev and Kramer‐Schadt 2021; Sibly et al. 2013). While most current simulation models focus on single species and single disturbances, such models also provide a promising tool to study species communities under multiple global change stressors (Pirotta et al. 2022). By applying the first community‐level individual‐based energetic model for terrestrial mammals (Szangolies, Gallagher, et al. 2024), we aim to assess the combined effects of habitat fragmentation and drought events on species coexistence using a small mammal community as a case study system. Our simulations address the following questions:

1. How do individual energy dynamics respond to extreme events, like droughts, and how do they mediate population persistence?
2. Does intermediate habitat fragmentation remain beneficial for species coexistence in communities when additional disturbances, such as droughts, occur?
3. What mechanisms allow for population persistence and high species richness under multiple global change effects?

## 2 Methods

### 2.1 Individual‐Based Metabolic Community Model

We used the individual‐based metabolic community model presented in Szangolies, Gallagher, et al. (2024). For a full description of model development, functioning, and validation, see the supplementary TRACE document (TRAnsparent and Comprehensive Ecological modelling documentation, Grimm et al. (2014), Data S2). Here, we only provide a summary of the key model processes.

**FIGURE 1:**
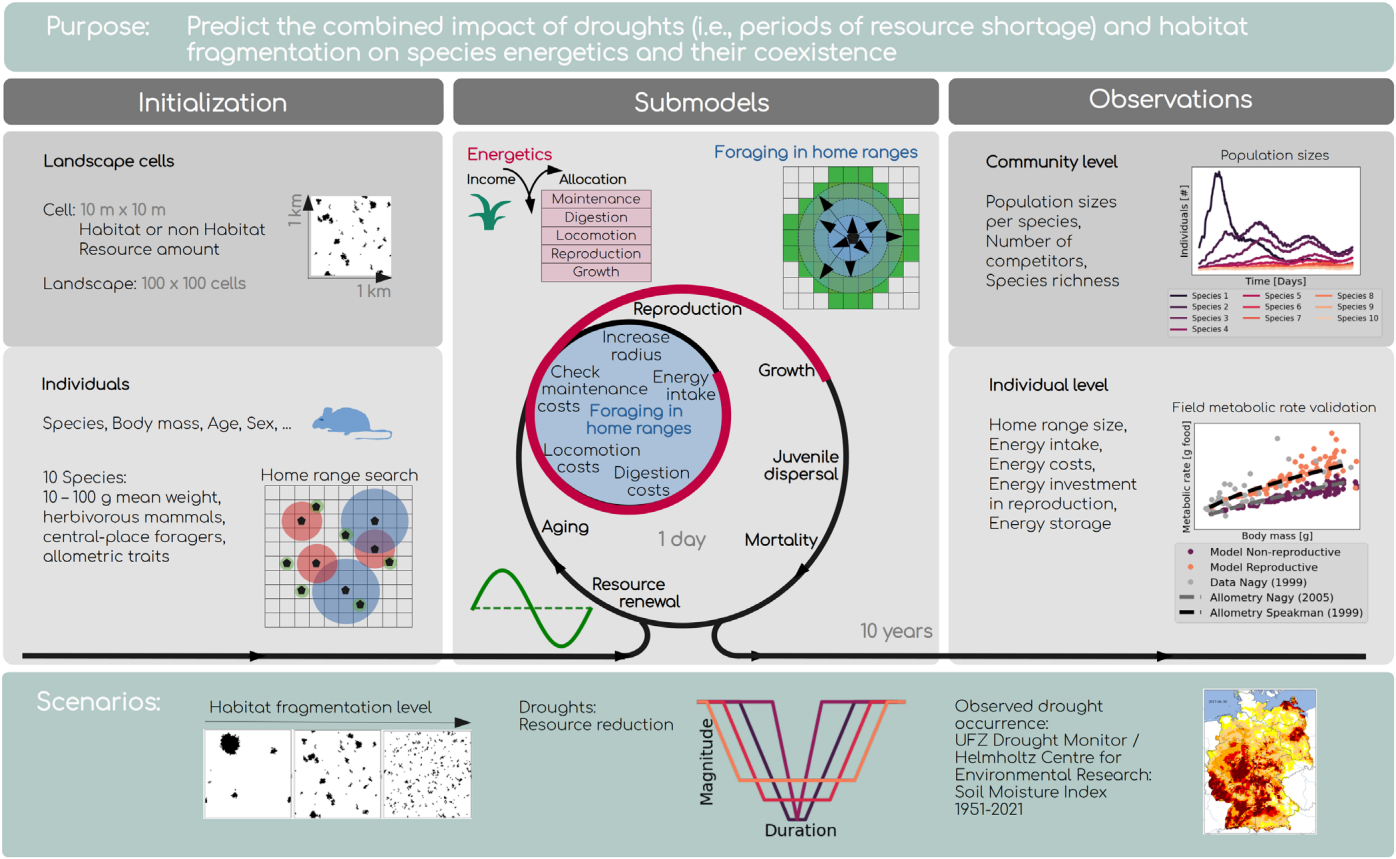
Visualisation of the energetic community model adapted from Szangolies, Gallagher, et al. (2024) using the vODD format (Szangolies, Rohwäder, et al. 2024). The purpose of the model is to investigate the impact of drought and habitat fragmentation on individual energetics and coexistence in a mammalian community. During model initialisation, landscapes with habitat and matrix patches are generated, and individuals of different species establish initial home ranges in the landscape. During daily timesteps, individuals forage in their home range to ingest energy (marked in blue), which they then invest into the metabolic processes of maintenance, digestion, locomotion, growth and reproduction (marked in red). At the end of a simulation run, typically 10 years, outputs on the individual and community level are generated. Several model outputs have been validated against literature data, such as the emergent field metabolic rate of individuals of different sizes. We simulate scenarios of varying landscape fragmentation and apply drought scenarios of different duration and magnitude. In addition, we use observed drought data from the German Drought Monitor (UFZ 2023).

The model simulates resource competition and survival of individual small herbivorous mammals in spatially variable landscapes (see Figure 1 formatted following the vODD model visualisation by Szangolies, Rohwäder, et al. (2024)). We simulate 10 species characterised by their body mass (10 g–100 g). Based on this body mass, several traits, such as maximum movement radius and energetic costs, are defined allometrically. The model simulates in daily timesteps how animals forage for food, compete for resources, invest energy in growth and reproduction, and ultimately survive or die within a landscape composed of habitat and non‐habitat cells. Each landscape cell is 10 m × 10 m in size, and in total, we simulate 1 km^2^. The cells that are considered habitat contain generic resources, which are renewed every day. In this application, we extended the model to allow for more natural resource variation by introducing a seasonal pattern, represented by a sinusoidal curve (green curve in Figure 1).

Central‐place foraging individuals move within home ranges that they first establish in initialisation or after juvenile dispersal. Home ranges are determined by evaluating potential core cells based on resource availability and energy needs. Individuals expand their search radius around a potential core cell until they find sufficient food to meet their needs and reach their maximum storage level (Lindstedt and Boyce 1985), reach their allometrically determined maximum home range size (Kelt and van Vuren (2001), reflecting daily time constraints), or exhaust their energy for movement. If the tested home range fails to cover their energetic needs, individuals try other potential locations up to a maximum defined number of trials, after which they are removed from the simulation. Once established, individuals forage within their home ranges every day by similarly increasing a search radius around the core cell. Home range sizes are thus adjusted daily according to resource availability and individual needs, and therefore vary between individuals, seasons, competition intensities, and an individual's energetic states. Resource availability is influenced by other individuals competing for food, with a new random foraging order of individuals every day. Though a slight advantage in foraging order is given to larger individuals and those with few conspecifics nearby (see details in Data S2).

Individuals ingest energy based on the available resources, an allometrically defined food share, and their daily needs. This energy is directly reduced by assimilation efficiency (Hendriks 1999) and digestive losses (Hindle et al. 2003). Following the energy allocation strategy proposed by Sibly et al. (2013), energy is then first allocated to processes necessary for survival (maintenance costs based on Savage et al. (2004)) and movement (locomotion costs). The locomotion costs are composed of postural costs for maintaining an upright posture (Halsey 2016) and incremental costs for moving forward (Calder 1996). If energy remains after fulfilling these costs, females can devote it to reproduction. During pregnancy and lactation, females attempt to cover the energy demands of their offspring based on offspring body mass and the additional cost of synthesising new flesh (Trebatická et al. 2007; Moses et al. 2008). From the first day of life, individuals grow according to allometrically defined growth curves (Gompertz functions for embryos, Ricklefs (2010), and von Bertalanffy functions after birth, Sibly et al. (2013)). If a mother cannot meet the energy needs of her offspring during pregnancy, she will abort the entire litter or, during lactation, lose one juvenile after the other. Once offspring become independent, their growth depends on securing enough energy for themselves. In general, if animals consistently consume less energy than they expend, they will deplete their energy stores and eventually die. Conversely, if they consume more energy than they expend, they store it as fat for later use (Lindstedt and Boyce 1985). Carrying this extra weight increases the energy cost of movement. To survive and reproduce, animals must balance their energy intake with their energy expenditure.

### 2.2 Fragmentation and Drought Scenarios

We simulated drought scenarios by introducing periods of resource scarcity, since droughts commonly decrease primary production and thus food availability for herbivores (Ciais et al. 2005). Thus, for a certain time, resources are reduced to a fraction of their normal availability. The magnitude of such a drought period can be very severe (resources 98% reduced), severe (resources 95% reduced), moderate (resources 90% reduced), or low (resources 80% reduced). We chose these levels to be similar to drought thresholds of soil moisture as used in the German Drought Monitor of the UFZ/Helmholtz Centre for Environmental Research (UFZ 2023; Zink et al. 2016). Although soil moisture may not directly translate to resources, it is used to calculate plant available water (UFZ 2023; Zink et al. 2016) and may be sufficient for our purposes of defining a drought period and magnitude. We simulated droughts of 3 days, 9 days, 22 days, and 57 days length (log scale, see exemplary scenarios in Figure 1). Each drought period included a 1‐day transition from normal resource availability to drought conditions at the beginning, and from drought conditions back to normal at the end of a drought. Thus, a 3‐day drought would have 1 day of reduction phase, 1 day of resources at the drought magnitude, and 1 day of recovery before resources return to normal. We also tested other lengths of transition periods and show exemplary results in Data S1. We applied one drought per simulation, always starting in the middle of a year in summer and after populations have reached a stable state.

We compared single‐species simulations, which included only one species per simulation run, with community simulations including individuals of 10 different species. For single‐species simulations, we applied the drought in the second year and ran simulations for a total of 3 years. For community simulations, we ran 10 simulation years with a drought in the fifth year, as stable states are reached later than in single‐species simulations.

While by default individuals during a drought follow the same behavioural rules as without drought, trying to forage as much as possible to meet their needs and fill their energy reserves, we introduced two additional scenarios where individuals adapt their behavioural decisions during periods of resource shortage. These alternative scenarios may reflect that individuals can react to resource stress and incipient starvation differently, possibly increasing or reducing movement (Gutman et al. 2007; McCue 2010). In the default simulation, individuals will increase their activity as a reaction to drought to maintain similar levels of food acquisition (Cornish and Mrosovsky 1965). As a second option, individuals largely maintain their activity level during food shortage by retaining their pre‐drought home range size. Hence, they do not expand their home range beyond the mean size observed in the 7 days prior to drought, irrespective of food availability. As a third scenario of adapted behaviour, individuals decrease their activity during drought to reduce energy expenditure and maintain energy balance this way. For this, we modified the foraging aim or motivation of individuals during a drought, so that individuals only forage until they have enough energy for survival, ignoring other processes. This can result in no foraging at all during the first day(s) of a drought when individuals have enough energy in their storage from before drought. This behaviour can resemble an energy conservation strategy, which several species apply (Vuarin and Henry 2014; Nowack et al. 2017).

In addition to drought, we applied three scenarios of habitat fragmentation and analysed their combined effects with drought conditions. The level of habitat fragmentation describes whether resource cells are relatively clumped together (low fragmentation), dispersed across the landscape (high fragmentation), or in between (medium fragmentation, see example landscapes in Figure 1). The total amount of habitat always remained the same.

Finally, instead of simulating a single drought per scenario, we applied time series of observed drought occurrences generated from historical topsoil moisture data from the German Drought Monitor of the UFZ/Helmholtz Centre for Environmental Research (UFZ 2023; Zink et al. 2016). From these data, we used 20 random locations across Germany and compared the time periods 1952–1962 and 2009–2019. Using the suggested drought thresholds, we defined drought periods in the time series and incorporated the resulting occurrences into our model. We show results for the 2% drought threshold (98% reduced resources in the model), 5% threshold (95% reduction), 10% threshold (90% reduction), and 20% threshold (80% reduction). Results for other combinations of drought thresholds in the data and drought magnitude in the model are presented in Data S1.

For each scenario, we ran 20 replicates. Data can be downloaded from Dryad (Szangolies et al. 2025).

## 3 Results

### 3.1 Drought Impacts on Populations

**FIGURE 2:**
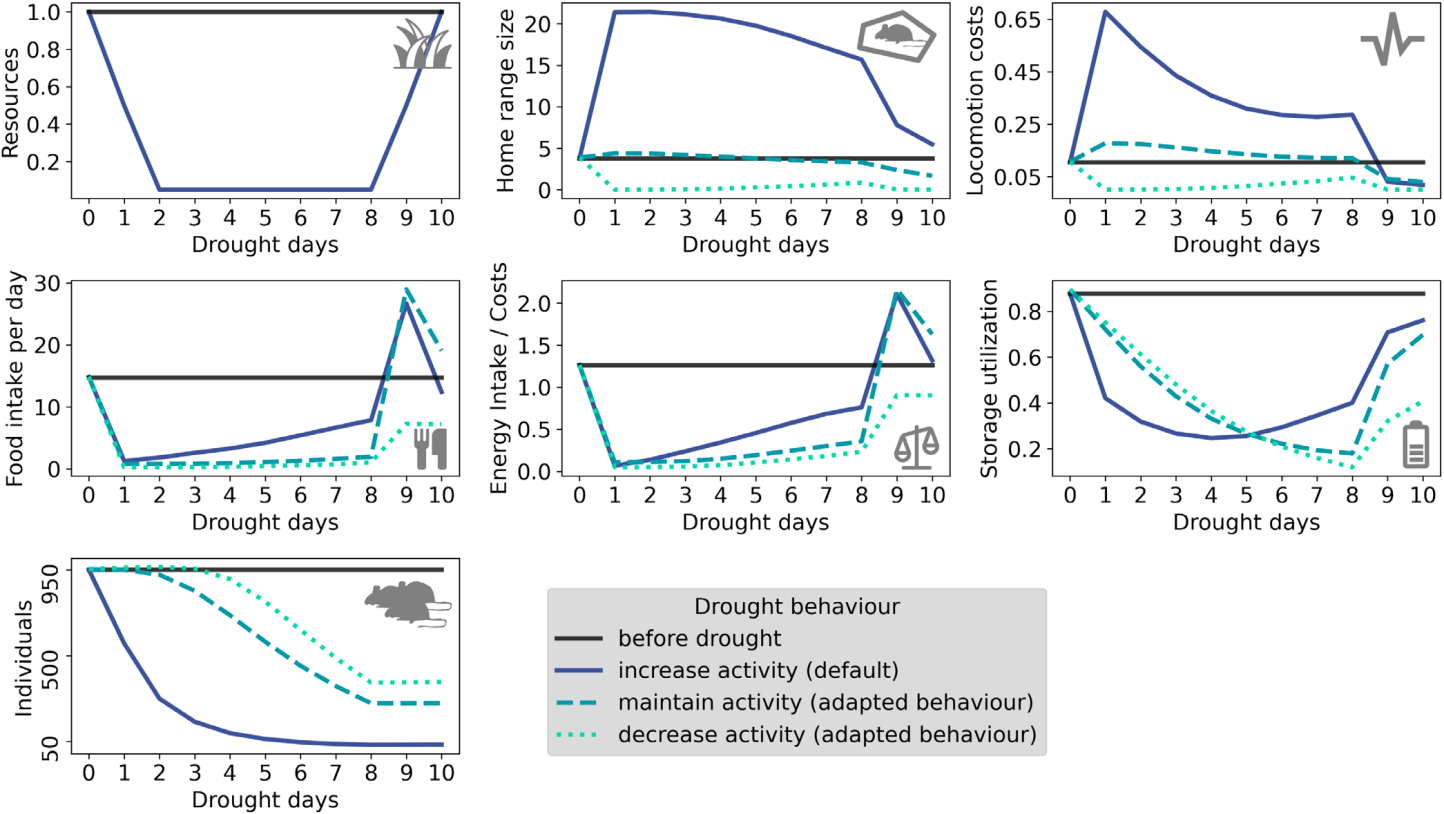
Mean species reaction to a drought of 9 days length with a resource reduction of 95% in a medium fragmented landscape as isolated populations. Three foraging strategies during drought are applied. The increase activity strategy is the default strategy with individuals foraging as much as possible. The maintain activity strategy means individuals keep the home range size similar to before the drought, and the decrease activity strategy reduces the foraging motivation of individuals which will then only try to survive ignoring other energetic processes. Shown are means of all individuals of all species over 20 replicates.

When a drought reduced the available resources, species usually increased their home ranges in an attempt to find sufficient food (Figure 2). The increase in home range size led to an increase in locomotion costs. Combined with a decrease in energy intake due to reduced food availability, this led to a direct decrease in energy balance (energy ingested versus energy expended). The shortfall in energy balance could be made up for by energy storage, which hence also reduced during a drought. While the increase in home range size was due to the model assumption that individuals always try to forage as much as possible, we saw the same or an even stronger decrease in energy balance and storage when we applied scenarios with reduced activity during a drought (Figure 2). The reason for this was an even lower energy intake when individuals did not increase their search radius. Overall, mortality due to starvation was high in all cases. It only occurred slightly later in the adapted behaviour scenarios since, with lower energy costs, individuals could live longer from their storage alone.

**FIGURE 3:**
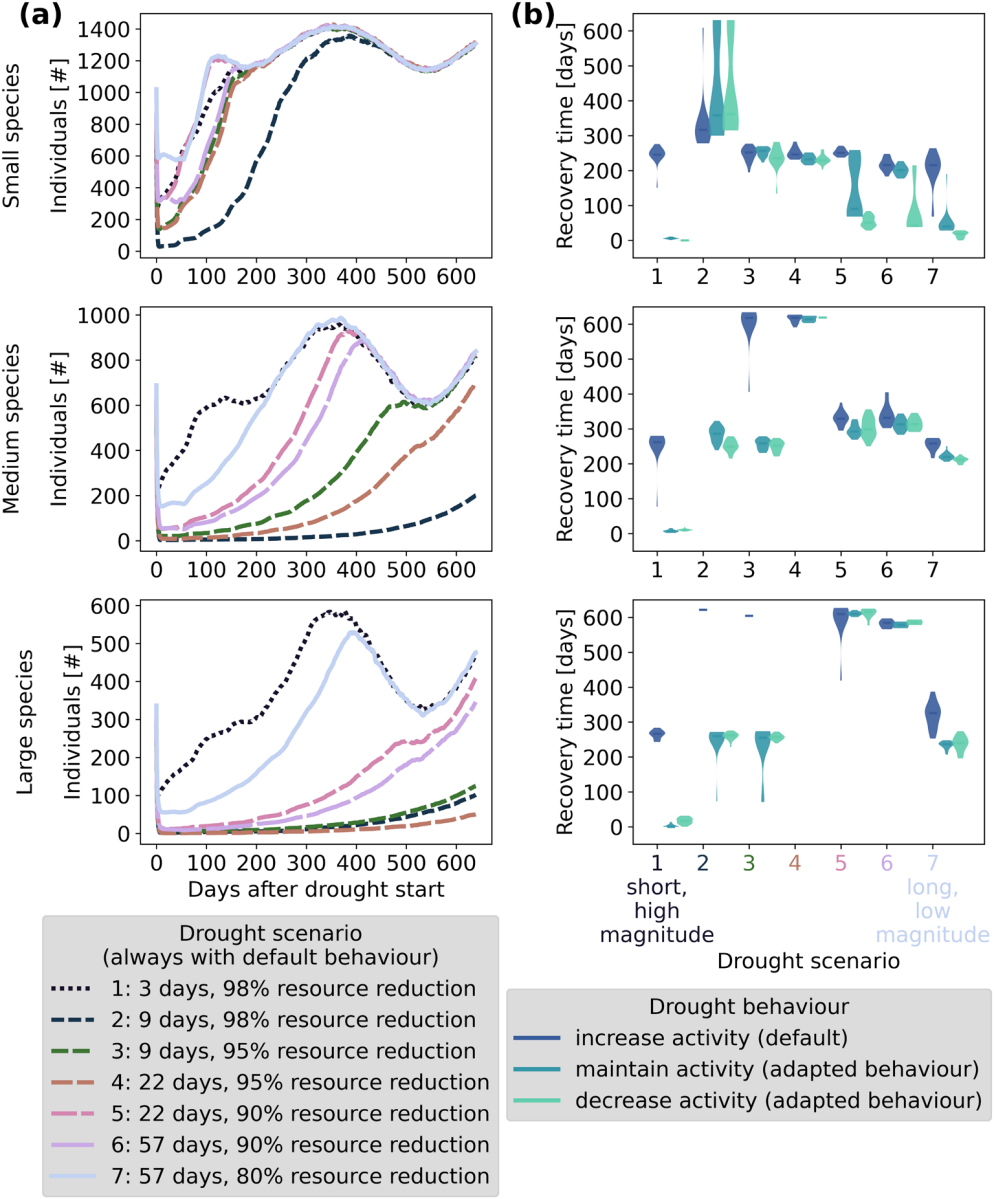
Reaction of population sizes to droughts of different lengths and magnitudes by the smallest (species 1), a medium‐sized (species 5), and the largest species (species 9). (a): Population dynamics after the drought. (b) Recovery time as the duration until populations reach their pre‐drought level. The recovery time (b) is shown for the default behaviour and two adapted drought behaviour strategies, while population dynamics (a) are only shown for the default behaviour (see Figure 2 for behaviour scenarios). Simulations were performed with medium habitat fragmentation and for single‐species populations in the landscape. Results are the mean of 20 replicates.

Nonetheless, populations were often able to recover after a drought. The time required for recovery varied depending on species and drought scenario (Figure 3). Severe droughts had a stronger impact, that is, longer recovery time, than long but less severe droughts, except when a drought lasted only 3 days. The recovery time and resistance of the populations showed similar patterns with often long recovery times and low resistance for high drought magnitudes (Figure 3b and Data S1 for resistance). In fact, drought magnitude seemed to have a stronger effect than drought length, as an increase in magnitude at constant length always increased recovery time (compare scenarios 2–3, 4–5, and 6–7 in Figure 3b), which was not necessarily the case for an increase in length at constant magnitude (compare scenarios 1–2, 3–4, and 5–6 in Figure 3b). When individuals adapted their behaviour to the drought period by maintaining or reducing activity, they recovered slightly faster in some drought scenarios. Overall, large species were affected by a drought for a longer period than smaller species, but they benefitted slightly more from reduced activity during drought.

### 3.2 Combined Effects of Droughts and Fragmentation on Populations and Coexistence

**FIGURE 4:**
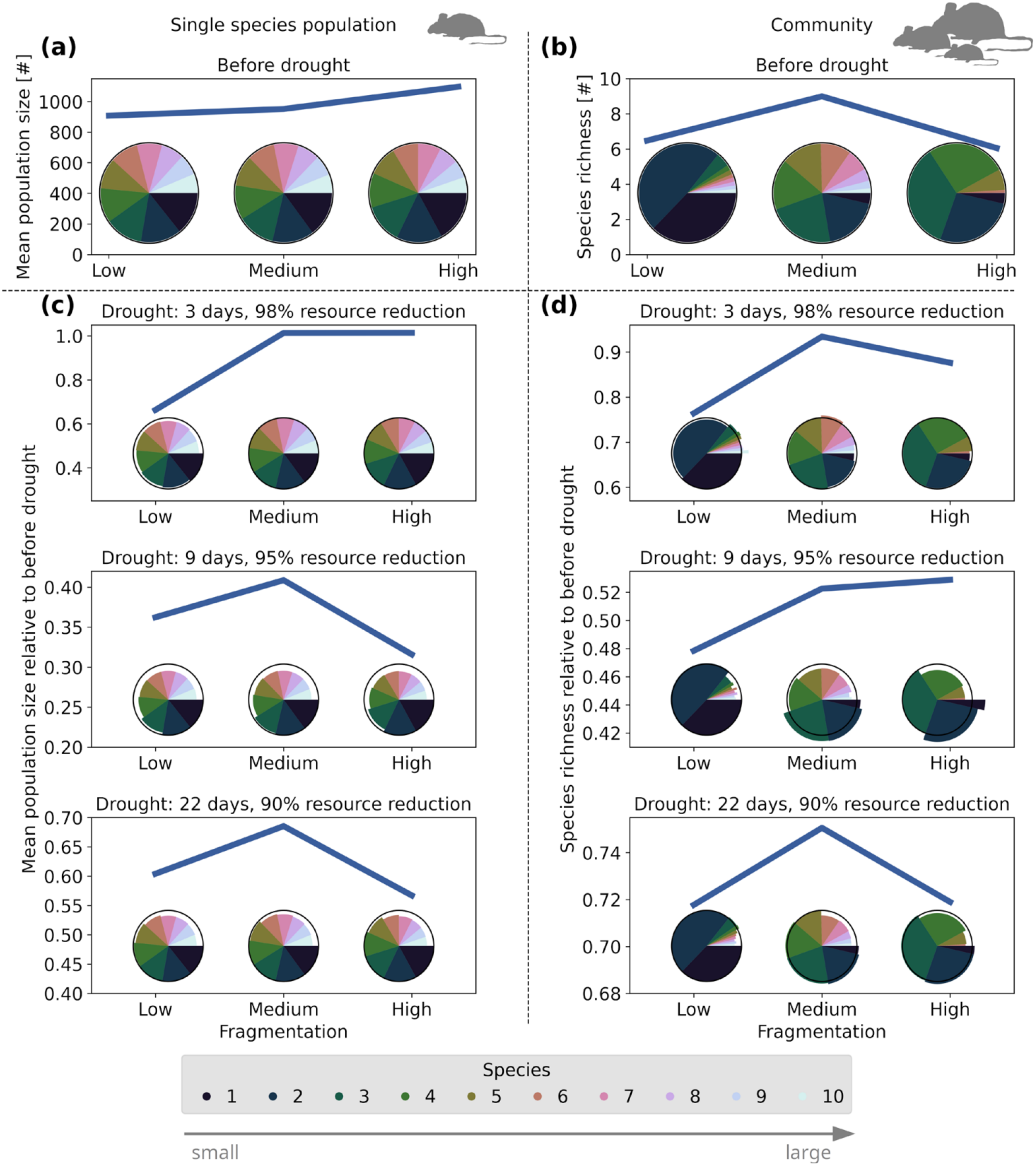
Effect of combined drought and fragmentation scenarios on mean population size, species richness, and community composition. (a) Mean absolute population size of all species alone in the landscape as line and mean relative population sizes as pie charts. Values measured after 3 years without any drought. (b) Mean species richness after 10 simulation years as line and mean community composition as pie chart without any drought. (c, d) Abundance of species (c) and species richness (d) 1 year after a drought relative to before drought, for single‐species simulations (c) and community simulations (d). Pie charts show mean species abundance relative to before drought as divergence from the black circle. Results are given for the default foraging behaviour during drought and are means of 20 replicates per scenario.

Incorporating differently fragmented landscapes to the drought scenarios showed that the landscape composition does, indeed, alter the recovery of populations 1 year after a drought (Figure 4). For very short droughts, population sizes and community composition changed most in low fragmented landscapes, compared to higher fragmentation levels. For longer droughts, all but the smallest species recovered best in medium fragmented landscapes in single‐species as well as community simulations (Figure 4c,d). Comparing drought scenarios in community simulations, larger species coped slightly better with short droughts, maintaining relatively stable population sizes. However, during longer droughts, their populations declined, allowing an increase of smaller species (Figure 4d). The overall highest species richness and population size at medium fragmentation was particularly interesting for single‐species simulations, as in these scenarios there was no benefit of medium fragmentation without drought (Figure 4a). In community simulations, medium fragmentation maximized species richness already before drought (Figure 4b), and also led to the least reduction in species richness with drought occurrence (Figure 4d). When species adapted their foraging behaviour to a drought period, medium habitat fragmentation still led to the lowest reduction in species richness due to similar reasons as in the default scenario (see Data S1). Further, under medium habitat fragmentation, species could persist over longer and more severe droughts before they went extinct compared to other fragmentation levels (see Data S1).

**FIGURE 5:**
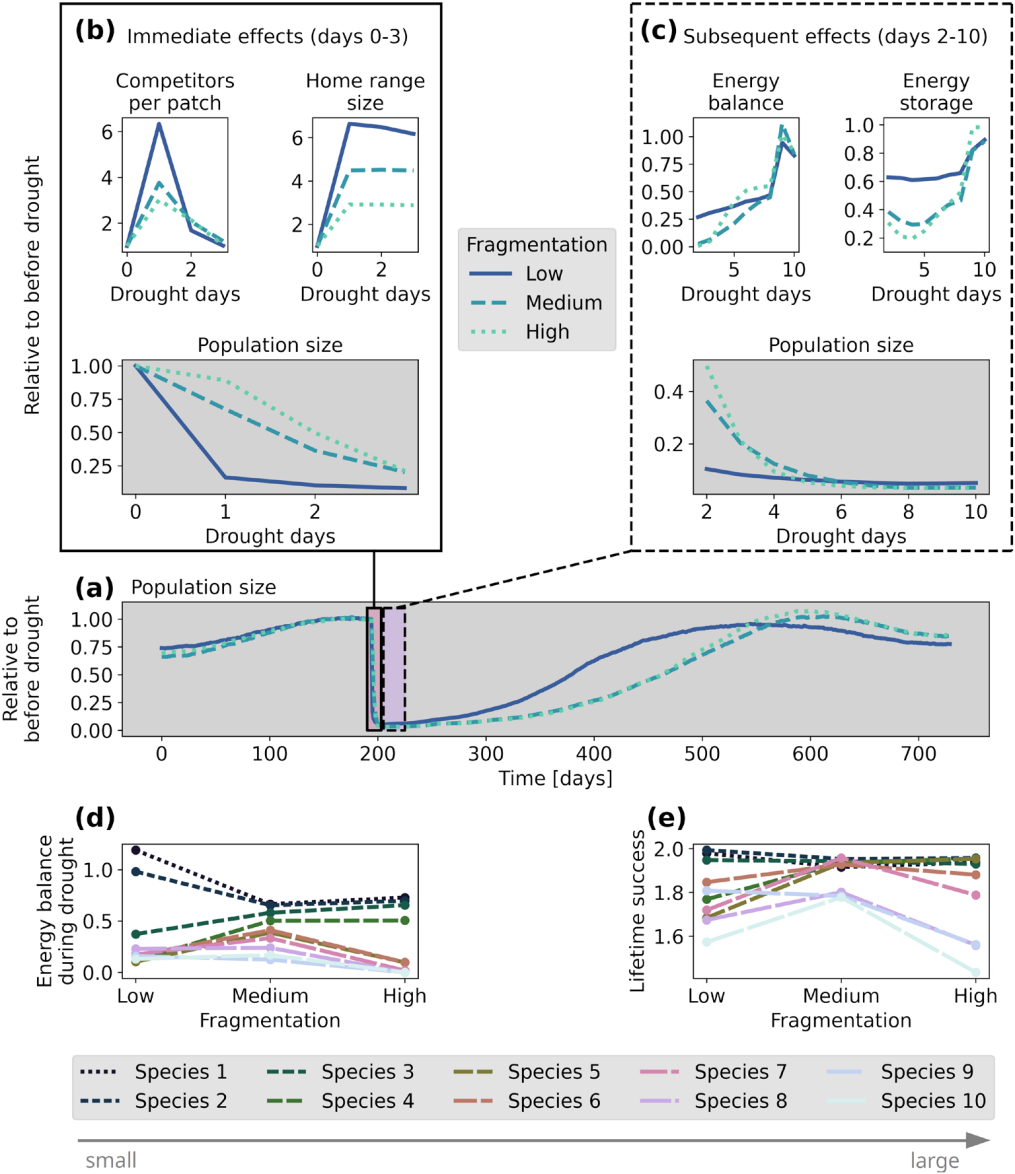
Mean species reaction to a drought in community simulations under different fragmentation levels with a drought of 9 days length and 95% resource reduction, and with default drought behaviour. (a) Relative population size over a longer simulation period. (b) Population size, number of competitors per foraging patch, and home range size at the beginning of a drought relative to before drought. (c) Population size, energy balance, and energy storage in the subsequent course of a drought relative to before drought. (d) Mean energy balance during the 9 days of drought for all species. (e) Mean lifetime reproductive success over the entire simulation for all species. Results are means of 20 replicates.

The reason why medium fragmentation best buffered against drought is not trivial (Figure 5). When a drought hit, species experienced resource scarcity. In low fragmented landscapes, where individuals lived close together in large habitat patches, they had a high number of competitors per foraging patch and thus less intake per foraging patch compared to other fragmentation levels (Figure 5b). Consequently, the individuals had to increase their home range size the most and thus had high locomotion costs. This effect induced high immediate mortality rates in the low fragmentation scenario, which was less the case in higher fragmented landscapes. Yet, in the subsequent course of a drought, individuals remaining in low fragmented landscapes could find more food at lower costs, leading to a lower reduction in their energy balance and storage compared to higher fragmentation levels (Figure 5c). The low energy balance and strong storage reduction in more fragmented landscapes induced higher mortality in these scenarios over the course of a drought. At medium habitat fragmentation, both the immediate and delayed effects were less severe compared to low and high fragmentation. Further, at medium fragmentation, species had the most similar energy balance over the course of a drought and the most similar lifetime reproductive success over the entire simulation (Figure 5d,e). This allowed for high coexistence in the long term in medium fragmented landscapes.

### 3.3 Observed Drought Timeseries

**FIGURE 6:**
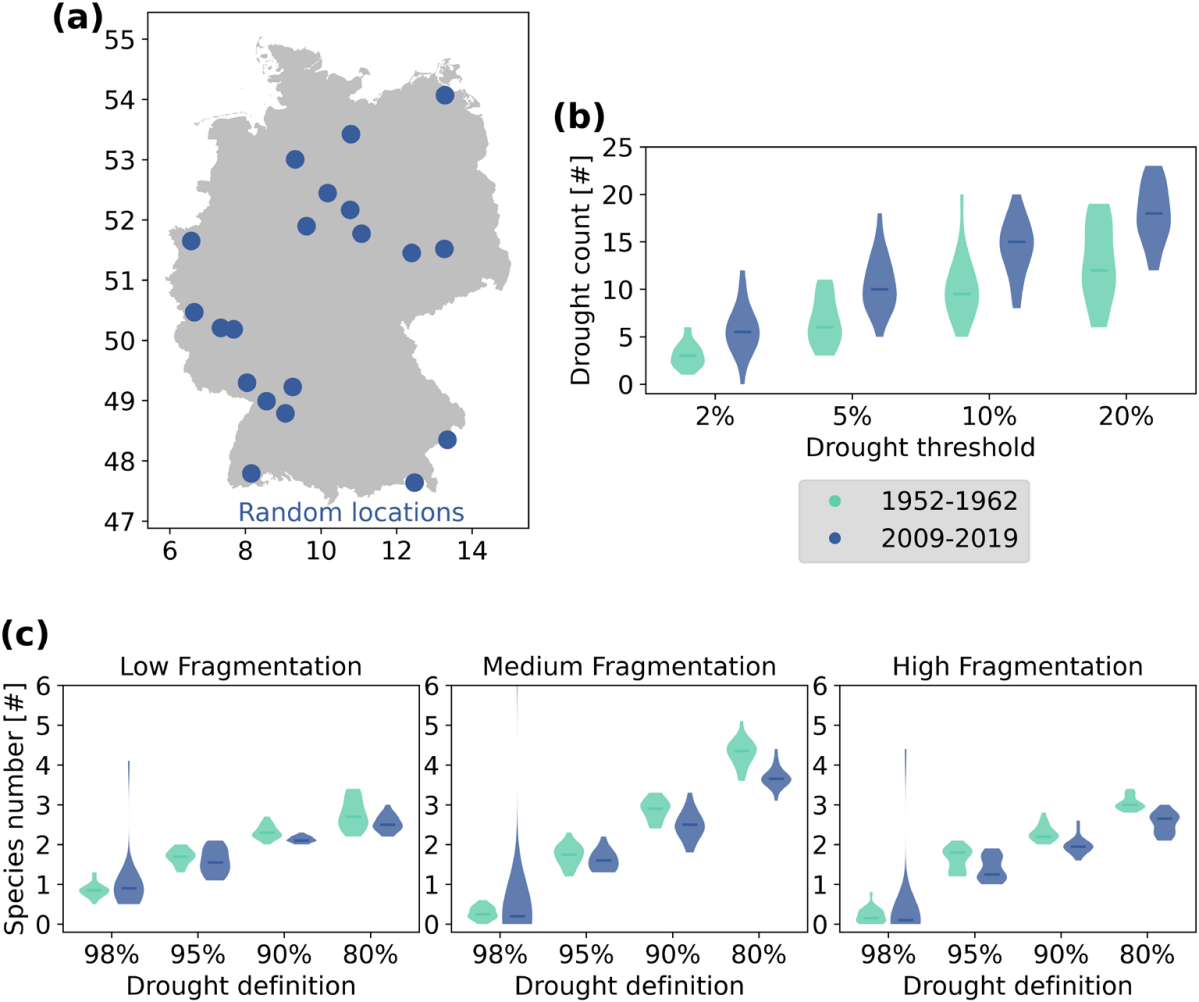
Simulation of observed drought timeseries from the German Drought Monitor of the UFZ/Hemholtz Centre for Environmental Research (UFZ 2023). (a) Locations of 20 random grid points from the German Drought Monitor that were used here. Map lines delineate study areas and do not necessarily depict accepted national boundaries. (b) Number of droughts at these locations in the two time periods 1952–1962 and 2009–2019, when droughts are defined using different thresholds of relative soil moisture, regardless of drought length (a drought can last from one to several days). (c) Number of species remaining after simulating the respective 10 years of drought occurrence defined by the German Drought Monitor data. We used a similar drought magnitude in the simulations as drought threshold for the data, that is, a drought definition of 98% means 98% resource reduction and 2% drought threshold in the data. We used artificial differently fragmented landscapes together with these drought timeseries and show the mean of 20 replicates.

Using the German Drought Monitor data of the UFZ/Helmholtz Centre for Environmental Research (UFZ 2023; Zink et al. 2016) to analyse the occurrence of droughts based on the topsoil moisture index for 20 random sites in Germany (Figure 6a), we saw an increase in the number of droughts from 1952 to1962 to 2009–2019 (Figure 6b). Naturally, the frequency of less intense droughts exceeded that of intense droughts. At one of the random locations, there were no very severe droughts recorded, resulting in exceptionally high species richness in simulation outputs (2009–2019, 2% drought threshold). The implementation of these drought timeseries in the model showed that in the time period from 1952 to 1962, more species would have remained after 10 years than in the period 2009–2019 when starting from similar initial species communities (Figure 6c). This was particularly the case for landscapes with high habitat fragmentation, while there was less of a difference for lower fragmented landscapes. Hence, drought effects were stronger in fragmented landscapes. Nonetheless, medium fragmentation generally supported the highest species diversity, before as well as after droughts occurred.

## 4 Discussion

By using an individual‐based metabolic community model to simulate the effects of global change, we observed how individual energy dynamics responded to extreme events, such as droughts. Individuals decreased both their energy balance and storage, which resulted from a reduction in energy intake and/or an increase in energy costs (Figure 2). The decreased energy balance and storage often led to a subsequent energy deficit for survival and thus high mortality. This effect partially depended on the amount of energy stored prior to a drought, which varied with habitat fragmentation level.

We could verify the benefit of intermediate habitat fragmentation for coexistence also in the presence of additional stressors such as droughts (Figure 4). In medium habitat fragmentation scenarios, species richness reduced the least during droughts, and populations recovered best. Thus, medium habitat fragmentation may best buffer the effects of drought.

Finally, we observed mechanisms by which individuals and species could cope with stressors. These underlying mechanisms for persistence under multiple stressors ranged from high stored energy reserves prior to drought to reduced local competition due to landscape features and behavioural adaptation to resource scarcity (Figure 5). Noticeably, similarity in energy balance among species and similar changes in energy balance during stress promoted coexistence. This was particularly visible in scenarios with moderate habitat fragmentation and drought occurrence.

### 4.1 Effects of (Multiple) Stressors

We found maximum coexistence at medium habitat fragmentation, both with and without droughts. For habitat fragmentation alone, similar evidence has been suggested by former field and modelling studies (Loke et al. 2019; Taranto et al. 2023; Dalkvist et al. 2011). However, there is still a debate around habitat fragmentation and its positive or negative effects for biodiversity (Fahrig et al. 2019; Fletcher Jr, Didham, et al. 2018).

The effect of droughts alone was negative in our simulations. Drought led to reduced energy balance and storage, and ultimately to high mortality, particularly in the case of high‐magnitude droughts. This corresponds well with empirically observed negative effects of drought on the population size of various species, including small mammals (Cárdenas et al. 2021; Goldingay et al. 2023), large herbivores (Knight 1995), and birds (Cruz‐McDonnell and Wolf 2016).

So far, little research has been conducted on the combination of habitat fragmentation and droughts. In our simulations, this combination has led to a stronger decline of species richness than any one of the stressors. While fragmentation alone reduced species richness by up to 30%, some drought scenarios caused declines of about 50%, and a combination of drought and habitat fragmentation reduced diversity by up to nearly 70%. Such a combined effect of habitat fragmentation and droughts has been observed in other taxa, including bees (Hung et al. 2021) and butterfly populations, where survival depended on both low fragmentation and low drought occurrence (Oliver et al. 2015). Similarly, Martin et al. (2006) argued that for snail kites, habitat fragmentation reduced the ability to find drought refugia and thus there was a combined negative influence of drought and fragmentation. Another example from arid landscapes showed that small carnivores could recover from periods with low precipitation but only in landscapes not already encroached by shrubs, that is, not highly fragmented (Blaum et al. 2007).

When simulating observed drought time series from different locations in Germany, again medium fragmented landscapes revealed the highest remaining diversity and highly fragmented landscapes showed the strongest negative effect of droughts on species richness. Since drought occurrence is increasing in Germany (Zink et al. 2016) and elsewhere (Vicente‐Serrano et al. 2022), it is important to design landscapes that are capable of buffering drought effects, that is, that are only intermediately fragmented.

Our results even indicate synergistic effects of droughts and habitat fragmentation, with impacts more severe than expected by summing the effects of each stressor in isolation. At high levels of habitat fragmentation, population sizes and species richness were relatively more reduced by drought than at medium levels of fragmentation. Such synergistic effects of multiple stressors have been found in several studies (Galic et al. 2018; Martínez‐Megías and Rico 2022). For example, the simulation experiment by Galic et al. (2018) highlighted severe combined effects, especially at higher organisational levels, for example, population‐, community‐, and ecosystem‐levels.

Mechanistically, in our simulations, this combined and interactive effect resulted from low energy storage in highly fragmented landscapes, which led to an increased starvation risk under drought conditions. Hence, habitat fragmentation, as a landscape change, amplified the sensitivity of individuals to drought. Conversely, drought stress, as a climate change effect, often intensified competition with individuals expanding their foraging range to overcome resource scarcity, as also described by Sergio et al. (2018). This was particularly problematic at low habitat fragmentation, where individuals lived in close proximity. Hence, drought occurrence affects the impact of landscape and land‐use change on biodiversity. This is in line with Schulte to Bühne et al. (2021), who described in a review how land‐use change can alter the exposure, sensitivity, and adaptive capacity of an ecosystem, putting it more at risk under additional climate change effects, and vice versa. Their proposed framework provides examples of processes driving these interactions and highlights the importance of studying combined effects of land‐use and climate change on species.

### 4.2 The Role of Energy Dynamics for Communities

To mechanistically better understand the impacts of stressors and global change effects, we must consider the biological processes that link environmental change to individual fitness and higher‐level dynamics. We suggest that at the individual energy level, the effects of different global change impacts and stressors can be well considered simultaneously, which is often difficult otherwise (Orr et al. 2020). On the one hand, different stressors may mechanistically affect similar energetic processes, such as energy intake, through altered food availability. On the other hand, stressors may have interactive effects through trade‐offs in energy investment and expenditure. In our simulations, droughts primarily affected energy intake, while fragmentation strongly affected energetic costs of locomotion. Additionally, energy storage was important for individuals to survive drought periods, but energy storage prior to drought depended on habitat fragmentation. Hence, the energy dynamics integrated these stressors through individual energy balance, which is closely tied to fitness (Brown et al. 2004). Integrating an energetic perspective therefore provides a promising avenue for studying the multitude of interacting effects of multiple stressors (Orr et al. 2020; Simmons et al. 2021). For example, Simmons et al. (2021), describing a variety of options for studying a combination of stressors, specifically acknowledge the potential of metabolism and physiology at the individual level to link different stressors.

Physiology and energy dynamics at the individual level scale up to the community level, not only through individual fitness but also through mediating coexistence. At medium habitat fragmentation, energy balance was most similar among species during drought, allowing for the highest resistance and recovery of the community. Hence, as suggested by Szangolies, Gallagher, et al. (2024), our results again indicate that similarity in energy balance and reproductive investment leads to the highest coexistence of species. Consequently, investigating energetics can increase the understanding of community coexistence (Brandl et al. 2023) also under multiple global change effects.

### 4.3 Context Dependence: Isolated Species Versus the Species Community

As many studies focus on individual species and it is often unclear whether responses are similar when species occur in communities, we used the energetic community model to simulate isolated single‐species populations as well as entire communities. We found that the ecological context in which species live is important for their response to stressors, with the benefit of medium habitat fragmentation present in both population‐ and community‐level simulations. However, in the single‐species simulations, pre‐drought population sizes were usually highest under high fragmentation, while mean energy balance and storage were highest under low fragmentation (see also Szangolies, Gallagher, et al. (2024)). Only after droughts did we observe a positive effect of medium fragmentation in single‐species simulations. In contrast, community‐level simulations showed the highest diversity at medium habitat fragmentation even before a drought since energy balance was most similar among species under these conditions (Szangolies, Gallagher, et al. 2024). Hence, we did not anticipate our findings of similar impacts on population persistence across different ecological levels under the combined stressors. Previous research has already highlighted the importance of considering biotic interactions when studying multiple stressors (Orr et al. 2020; Thompson et al. 2018; Turschwell et al. 2022). For example, Turschwell et al. (2022), using a mathematical model, showed that the effect of multiple stressors on seagrass varied depending on whether a population submodel or a full consumer‐resource model was used. He et al. (2023) identified a clear gap in the research of biotic interactions under multiple stressors in the case of freshwater systems impeding the development of management strategies for conservation. Generally, considering how stressors affect the different organisational levels from individuals to populations and communities may be key to understanding combined impacts on biodiversity (Simmons et al. 2021).

Zooming in on individual species, we see similar effects of drought across species of different sizes. Larger species benefitted slightly from their relatively greater capacity to store energy. This was particularly evident when considering scenarios with behavioural adaptation of reduced movement, where large individuals could survive longer on storage energy alone. In line with this, Rymer et al. (2016) suggested that larger species are more likely to endure periods of stress, while smaller species are more likely to evade if possible. However, if droughts last longer than stored energy can cover, large species may be more vulnerable due to their higher absolute energy requirements for survival (see also Wato et al. (2016)). In our simulations, this high energy demand led to markedly increased movement of larger species, especially in highly fragmented landscapes. In comparison, small species require less energy to survive, and due to their faster life history, they can recover more quickly after a period of stress (Stark et al. 2004). Small species may hence even benefit from reductions in the population size of other species due to a stressor, as they are able to recover and colonise the free space first.

### 4.4 Model Limitations and Extensions

Two specific stressors were investigated here, but many other global change effects may interact with these. With some adjustments, our model would allow the integration of other global changes, such as habitat loss or changes in the seasonality of resources. Addressing temperature changes more directly would prerequisite an integration of thermoregulatory energy costs and behavioural alterations, which could be done in the energetic part of the model. In addition, the findings for droughts, simulated here as periods of reduced resources, may be relevant for other disturbances which cause temporary resource shortages as well, such as pesticide effects or flooding. Furthermore, droughts could be simulated in a more detailed and realistic manner. We have used soil moisture from the German Drought Monitor to directly approximate available resources, which we believe is sufficient for our purposes. However, a more process‐based implementation of how soil moisture influences plant growth and seed production could be realised. Alternatively, data on plant‐available water, which takes into account soil pore volume, could be used instead of soil moisture (UFZ 2023).

For the effect of droughts, we only considered a reduction in resources, ignoring other effects such as drinking water restrictions. However, some species may not need to drink, gaining all their water from their food (Nagy 1994). Similarly, we did not directly consider species‐specific adaptations to periods of resource shortage, such as torpor or hibernation (Nowack et al. 2017; Vuarin and Henry 2014). However, we simulated additional drought strategies with reduced activity and hence reduced costs, and these did not significantly alter mortality rates or coexistence outcomes. Although we have tested such different behavioural strategies which occur in response to drought or other stressors (Gutman et al. 2007; McCue 2010), the overall consequences for population size and energy dynamics remained similar. Hence, while foraging strategy may depend on specific species or climatic condition, the qualitative effects of drought are likely to remain similar.

While we applied observed drought occurrence data from specific sites, we still used artificially fragmented landscapes unrelated to these sites. This may be useful for adapting local landscapes in the future, but most importantly it showed that for almost all situations, medium habitat fragmentation led to the highest species richness after 10 simulated years with drought occurrence.

### 4.5 Conclusion and Implications

In conclusion, by applying an energetic community model to simulate population and community responses to the combined global change effects of drought and habitat fragmentation, we identified a mechanistic basis for better understanding how these drivers affect population and community dynamics through changes in home range size, energy balance, and energy storage. We suggest that the combined impacts of global change effects should not be ignored, and mechanistic energy models, such as the one we applied here, can be valuable tools for understanding the interaction of effects. Observed environmental data can be integrated into the modelling, as we have done with the German Drought Monitor data, to generate predictions and tailored conservation strategies. Given the synergistic negative effects of stressors that we have found, it will become even more important to counteract each stressor as much as possible. For example, while it may be challenging to prevent droughts, it could be feasible to design landscapes that are less fragmented or to prevent further fragmentation. While we are not suggesting that existing large habitats should be fragmented, we found that an intermediate level of habitat fragmentation was most beneficial for diversity. This points to the relevance of smaller habitat fragments that can contribute to an overall moderately fragmented landscape providing important functions. Hence, the effects of stressors are not always straightforward, and a mechanistic understanding of these effects is needed. To improve predictions and conflate the consequences of multiple global change effects on species and biodiversity, it is promising to study energy dynamics as a unifying measure. An additional benefit may be that conservation measures could directly address the energetic consequences of the impacts when it is not possible to prevent stressors themselves. Examples include providing additional food resources to increase energy intake, reducing movement costs by providing easily navigable corridors, or designing refugia that are protected from stressor effects. Such measures could be used to equalise the energetic effects of stressors and thus protect the energy balance in species communities. To effectively identify such interventions, it is crucial to understand the energy dynamics of species in their community and predict their responses to global changes.

## Author Contributions

**Leonna Szangolies:** conceptualization, data curation, formal analysis, investigation, methodology, software, validation, visualization, writing – original draft, writing – review and editing. **Cara A. Gallagher:** conceptualization, investigation, methodology, supervision, writing – review and editing. **Florian Jeltsch:** conceptualization, funding acquisition, investigation, methodology, project administration, supervision, writing – review and editing.

## Conflicts of Interest

The authors declare no conflicts of interest.

## Supporting information


Data S1.

Data S2.

## Data Availability

The data and code that support the findings of this study are openly available in Dryad at https://doi.org/10.5061/dryad.r7sqv9spk and Zenodo at https://doi.org/10.5281/zenodo.15131666, respectively.
