# Supplementary material for: Intermediate Habitat Fragmentation Buffers Droughts: How Individual Energy Dynamics Mediate Mammal Community Response to Stressors
Source: Glob Chang Biol. 2025 May 14;31(5):e70224. doi: 10.1111/gcb.70224 (PMC12077070; doi:10.1111/gcb.70224)
Supplement: Supplementary file 1 — Data S1. [file GCB-31-e70224-s002.pdf]

Supporting information 1: Additional Results

Intermediate habitat fragmentation buffers droughts: How individual energy dynamics mediate mammal community response to stressors

Resistance to drought

Additionally to the recovery time shown in Fig. 3 of the manuscript, there are other stability metrics describing population dynamics in reaction to a disturbance such as a drought. One of them is resistance, i.e., the maximum reaction of the population size in response to a drought relative to before. Resistance was highest for the very short drought in scenario 1, where the recovery rate, i.e., time to recovery was usually relatively low (Fig. S1.1). Resistance was usually lowest for the very intense drought in scenario 2, where recovery took relatively long or there was no recovery at all. Additionally, resistance was usually slightly higher in the scenarios with adapted drought behaviour. Adapted drought behaviour of large species particularly led to higher resistance and lower recovery time for short droughts since these species could live from their storage alone for this short period of time, but there was less difference between behaviours with longer droughts.

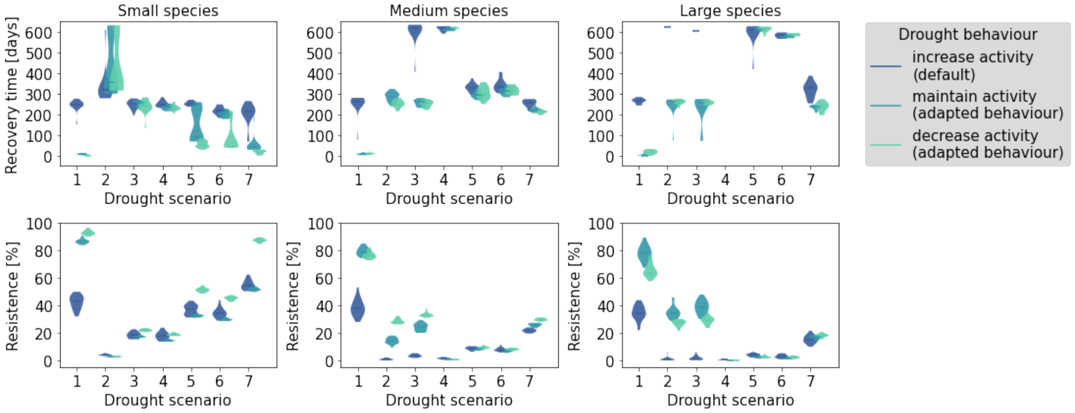

Figure S1.1: Recovery time and resistance of the smallest, a medium-sized, and the largest species when alone in the landscape in reaction to different drought scenarios and with different drought behaviour strategies. The increase activity strategy is the default strategy with individuals foraging as much as possible. The maintain activity strategy means individuals keep the home range size similar to before the drought, and the decrease activity strategy reduces the foraging motivation of individuals which will then only try to survive ignoring other energetic processes. The drought scenarios are: 1: 3 days with 98% resource reduction; 2: 9 days with 98% resource reduction; 3: 9 days with 95% resource reduction; 4: 22 days with 95% resource reduction; 5: 22 days with 90% resource reduction; 6: 57 days with 90% resource reduction, 7: 57 days with 80% resource reduction. The recovery time is the time until a population is back to before drought level (high recovery time means long time to recover), and resistance is the relative reaction of population size to drought (high resistance means small population change). We performed 20 replicates per scenario.

Fragmentation effect with other drought strategies

While the drought strategies slightly changed the behavioural reaction of individuals to a drought, the benefit of intermediate habitat fragmentation remained present (Fig. S1.2). The largest difference of the additional scenarios to the default strategy can be seen for a drought length of nine days. This may be the maximum time that particularly large species can overcome solely by their storage when not moving too much. Nonetheless, even for this scenario, the species richness was higher in medium fragmented landscapes compared to low or high fragmentation. This was the case because of the same effects as in the default scenario, they just occurred slightly delayed and partially less pronounced (Fig. S1.3). With decreased activity during drought, we saw that low fragmentation still led to the highest number of competitors and largest home range size compared to other fragmentation levels, although the numbers were now lower than before drought and the effect only occurred after a few days of drought. Still, this led to a more steep initial reduction of the population size than in scenarios with higher fragmentation levels. In the subsequent course of the drought, there was most reduction of energy storage in the high fragmentation scenario, which then led to the highest mortality in this scenario, although this effect was only slightly pronounced. Similar to the default scenario, at medium habitat fragmentation, we saw most similar energy balance during the drought and similar lifetime reproductive success, allowing for most coexistence of species.

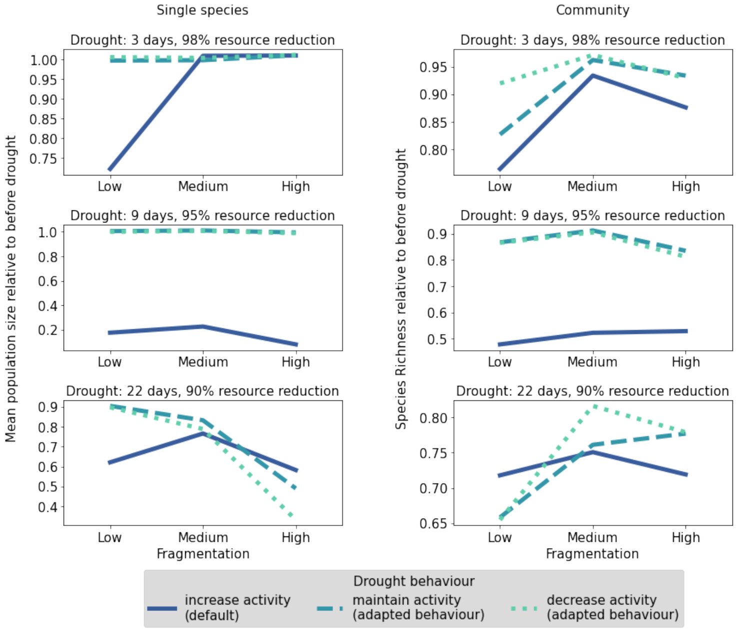

Figure S1.2: Mean population size one year after drought relative to before drought of all species alone in the landscape (left) and mean species richness relative to before drought in community simulations (right) for three scenarios of behaviour during drought, different drought scenarios and in differently fragmented landscapes. For details on the behavioural scenarios see Fig. S1.1. Results are means of 20 replicates per scenario.

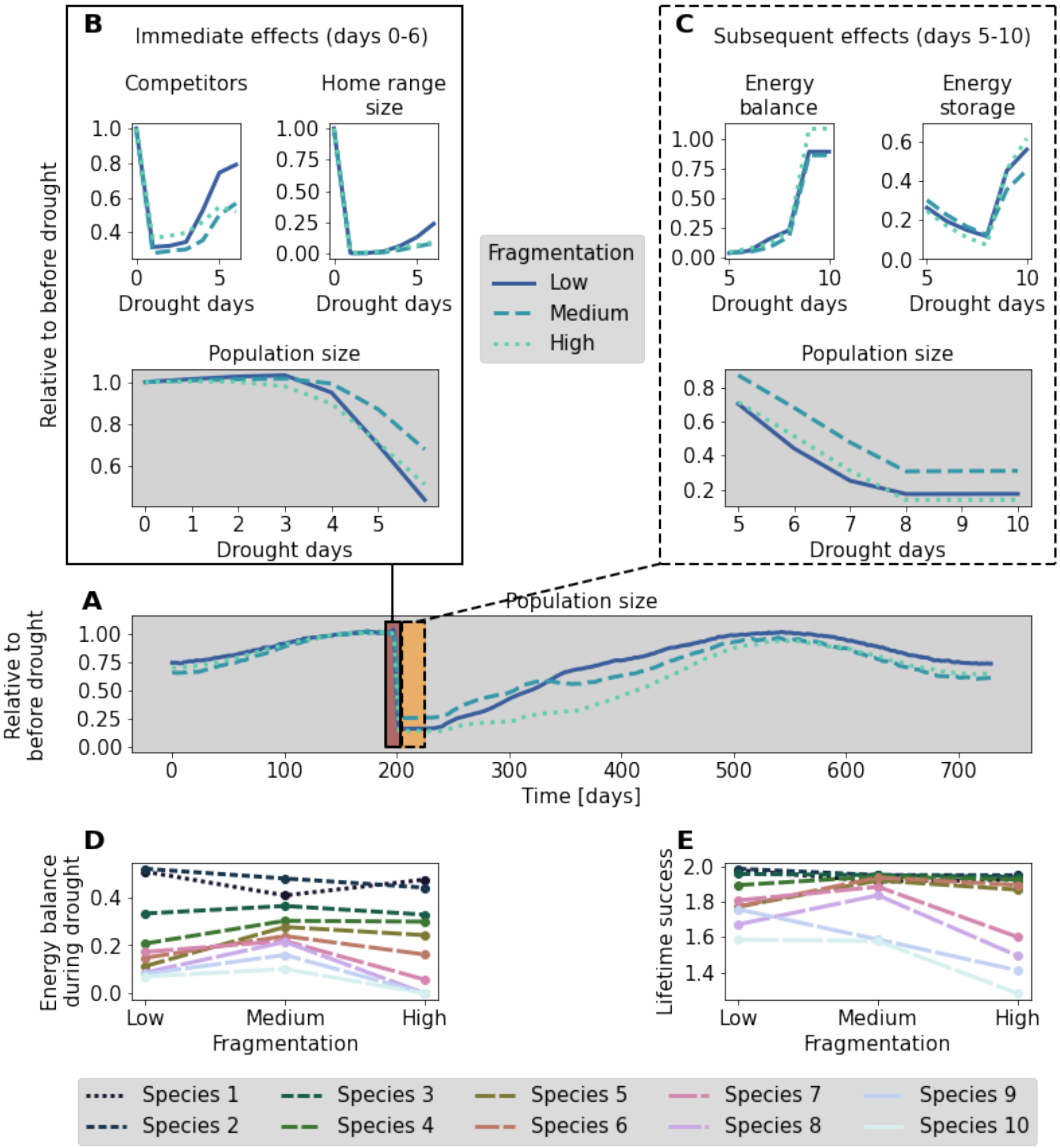

Figure S1.3: Mean reaction of all species to a drought of 9 days length with 95% resource reduction in community simulations under different fragmentation levels and for a drought strategy with decreased activity, i.e., foraging only to survive. A: Relative population size over a longer simulation period. B: Population size, number of competitors per foraging patch, and home range size at the beginning of a drought relative to before drought. C: Population size, energy balance, and energy storage in the subsequent course of a drought relative to before drought. D: Mean energy balance during drought for all species. E: Mean lifetime reproductive success over the entire simulation for all species.

Extinction thresholds

By simulating, with a full-factorial design, several drought lengths on a log scale (3 days, 5 days, 9 days, 22 days, 57 days, 150 days), all drought magnitude levels (98%, 95%, 90%, 80% resource reduction) and the three different fragmentation levels, we evaluated under which conditions species finally went extinct (if ever). When species were alone in the landscape, they tolerated longer and more intense droughts, than when they had to compete with other species (Fig. S1.4). In those single-species simulations, extinction only occurred with intense droughts (98% or 95% resource reduction). Conversely, in community simulations, extinction also occurred with less intense drought (90% and 80% resource reduction), particularly for large species. Generally, larger species already went extinct under less intense conditions than smaller species. Overall, most species survived more intense drought conditions when they lived in landscapes with intermediate habitat fragmentation, than if there was low or high fragmentation. Hence, we again saw the benefit of medium habitat fragmentation here, buffering the effects of droughts the best.

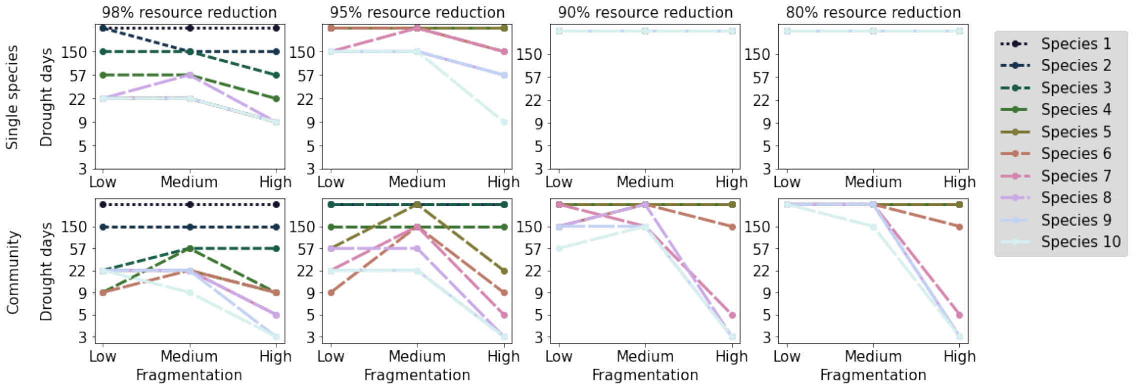

Figure S1.4: Extinction thresholds of species in all fragmentation and drought scenarios with default behaviour during drought. Points are the first scenario in which a species went extinct in all replicates, i.e., if there is a point at 98% resource reduction during 22 drought days (e.g., species 10, low fragmentation), then the species still survived a 98% drought of a length of 9 days, but not of a length of 22 days. Points above the 150-days line indicate that none of the scenarios led to an extinction.

Droughts with five days transition phase

When it took more time for the resources to reduce from a normal level to the drought magnitude level and to return to the normal level, this extended the entire drought period. By default, we simulated one day transition phase at the beginning and end of a drought, but as comparison, we additionally simulated five days transition phase. This longer transition phase led to a more slowly increase in home range size and locomotion costs and a more slowly decrease in food intake and energy balance, storage, and population size (Fig. S1.5). However, the intensity of the reaction was the same as soon as the drought magnitude was reached. At the end of a drought, home range size already started to reduce a lot as soon as resources started to increase again due to low competition with less population size and higher food availability. Overall, the drought reaction was pretty similar despite those different transition phases. Consequently, community results also mostly aligned with the default scenario (Fig. S1.6). Only in the intermediate drought length and magnitude scenario, the benefit of medium fragmentation was not as visible, but in other drought scenarios it was still present.

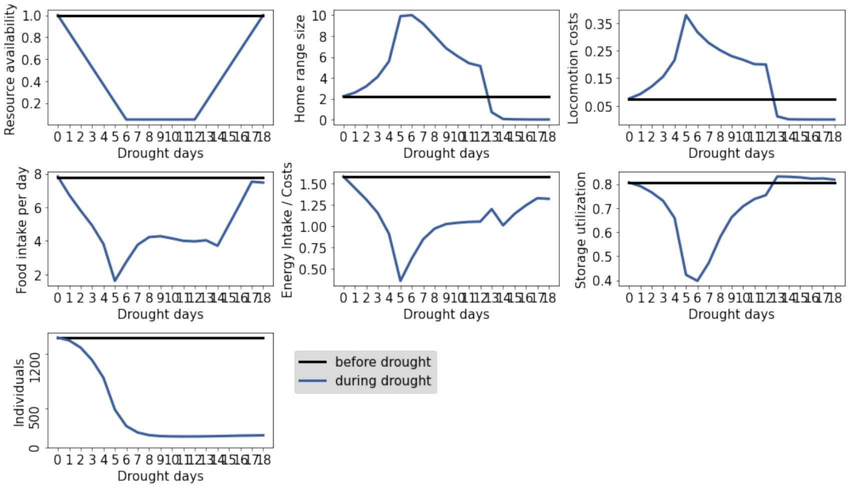

Figure S1.5: Exemplary reaction of species 1 when alone in the landscape to a drought of 95% resource reduction and 17 days length including 5 days transition phase at the beginning and end of the drought in a medium fragmented landscape. Shown is the mean over all individuals foraging with the default strategy.

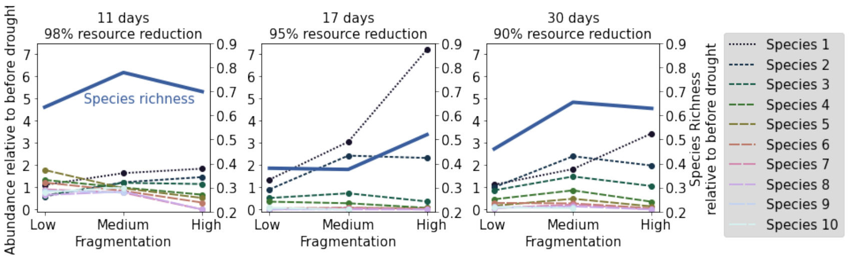

Figure S1.6: Mean abundance of species in the community one year after drought relative to before drought and mean species richness (thick dark blue line) in three exemplary drought scenarios that include a transition phase of five days at the beginning and end of the drought.

Observed droughts with other thresholds and magnitudes

We used the drought data from the German Drought Monitor (Zink et al., 2016) to define drought periods with different thresholds. While initially we used a similar drought magnitude as drought threshold, we additionally did a full-factorial design here, combining all drought thresholds with all drought magnitudes of resource reduction (Fig. S1.7). A reason for this was that the soil moisture defining a drought in the drought monitor data may not directly align with the resource level during a drought. Nonetheless, the results of all possible combinations again showed, that usually in the later time period 2009-2019 less species remained in the simulation than in the earlier time period 1952-1962. With low habitat fragmentation, there was usually less of a difference between the time periods than with higher fragmentation, but the highest species richness was present with medium habitat fragmentation. Comparing drought thresholds, species richness was lower with the higher drought thresholds since a low threshold defining drought occurrence resulted in fewer droughts than a high drought threshold.

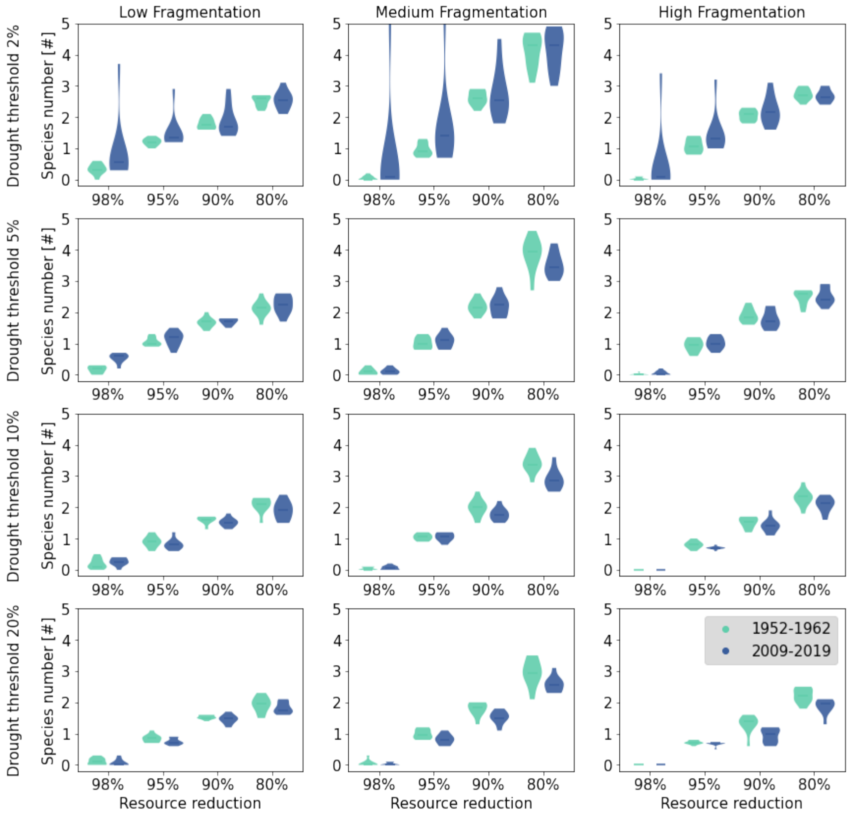

Figure S1.7: Number of species remaining after simulating ten years of drought occurrence defined by the German Drought Monitor data (Zink et al., 2016) at ten random locations in Germany and during two contrasting time periods. The definition of drought occurrence from the soil moisture data was done using four proposed thresholds (2%, 5%, 10%, 20%) and drought magnitude was defined using similar levels (98%, 95%, 90%, 80% resource reduction). We simulated a full-factorial design of drought thresholds and magnitudes and show means of 10 replicates.
