# Supplementary material for: Intermediate Habitat Fragmentation Buffers Droughts: How Individual Energy Dynamics Mediate Mammal Community Response to Stressors
Source: Glob Chang Biol. 2025 May 14;31(5):e70224. doi: 10.1111/gcb.70224 (PMC12077070; doi:10.1111/gcb.70224)
Supplement: Supplementary file 2 — Data S2. [file GCB-31-e70224-s001.pdf]

Supporting information 2: TRACE

Intermediate habitat fragmentation buffers droughts: How individual energy dynamics mediate mammal community response to stressors

This TRACE document describes the development, functioning, and evaluation of the dynamic energetic mammal community model first described in Szangolies et al. (2024). Specific adaptations for the present study are added in blue.

Contents

1 Problem formulation 2

2 Model description (ODD) 3

3 Data evaluation 13

4 Conceptual model evaluation 15

5 Implementation verification 16

6 Model output verification 17

7 Model analysis 18

8 Model output corroboration 21

1 Problem formulation

This TRACE element provides supporting information on: The decision-making context in which the model will be used; a precise specification of the question(s) that should be answered with the model, including a specification of necessary model outputs; and a statement of the domain of applicability of the model, including the extent of acceptable extrapolations (Grimm et al., 2014).

Summary: Individuals need to maintain a positive balance between incoming energy and energy costs in order to survive and reproduce. Their energy income and costs are influenced by landscape characteristics and competition with others. Thus, energetics can provide a link between the environment, individuals, and community dynamics. To analyze this link and to illustrate the effect of habitat fragmentation on energetics and community diversity, we developed an individual based metabolic community model. We parameterized the model for a small mammal community, but as it is largely based on allometric relations, it could also be applied to other species groups. We argue that the results can be extrapolated to general theory.

Environmental change is affecting biodiversity worldwide. To predict future diversity, we need to understand the mechanisms of community coexistence and how they interact with environmental change. One proposed mechanism of coexistence is movement (Schlägel et al., 2020). Recently, metabolic traits have also been suggested to explain community assembly (Brandl et al., 2023). Combining these mechanisms, we developed a community model that explicitly incorporates movement and detailed energetics. To our knowledge, our model is one of the first to combine these elements and scale up to the community level.

We use the model to simulate differently fragmented landscapes in which individuals move to find food. In this process, individuals compete for the food. Depending on their energy intake, individuals can sustain their energetic processes, such as maintenance, digestion, movement, growth, and reproduction. The energy balance, i.e., incoming versus outgoing energy, determines the success of an individual, a species and the coexistence of multiple species. This energy balance is affected by competition and by the energy available in the landscape, i.e., the landscape scenario.

With this model, we aim to show how energetics can help to understand coexistence. To do this, the model outputs include population sizes over time as well as individual movement patterns and energetics. The results show that energetics provide a direct link between the environment and the status of individuals. Furthermore, energetics define the competitive ability of species and where some species have advantages over others. Although we have simulated a small mammal community, we argue that the results can be extrapolated to other species groups, as food competition is an important interaction between species of various taxa. Furthermore, the simulation of species in our model depends only on body mass, which can be adapted for other species groups. We suggest that the use of energetics in general, will provide new insights into community theory.

These new theoretical insights can also improve predictions of biodiversity under environmental change. We simulate differently fragmented landscapes in order to find landscape compositions that are most beneficial for biodiversity. To do this, the model reports the species number over time for different landscape scenarios, so that they can be compared. The findings can be used in applied conservation, as landscapes may be designed accordingly. Additional to fragmentation issues, the model can also be applied to study, i.e., changes in resource availability and seasonality to incorporate climate change effects. However, as the model is rather theoretical, some further validation of the results would be recommended before application in practical conservation.

Additionally to habitat fragmentation, multiple other global change effects threaten biodiversity. It is hence important to study multiple stressors simultaneously as they may have combined effects. Energetics allows to study several global change effects in combination as it forms a link between different impacts through the energy budget and can thus conflate the diverse global change effects. One currently relevant climate change effect are droughts, which also occur in already fragmented agricultural landscapes. Hence, understanding the combined effects of habitat fragmentation and droughts as example for a multiple stressors effect on biodiversity is one aim of the present application of the model.

2 Model description (ODD)

This TRACE element provides supporting information on: The model. It provides a detailed written model description. For individual/agent-based and other simulation models, the ODD protocol is recommended as standard format. For complex submodels it should include concise explanations of the underlying rationale. Model users should learn what the model is, how it works, and what guided its design (Grimm et al., 2014).

Summary: Here we provide a model description following the classical ODD concept (Overview, Design concepts, and Details, Grimm et al., 2020). This model extends the previous individual-based home range community model (Buchmann et al., 2011; Rohwäder & Jeltsch, 2022; Szangolies et al., 2022; Teckentrup et al., 2018) by including details on individual energetics (based on Sibly et al., 2013). The model was implemented in Netlogo 6.0.4. A second version of the model, and data analysis and visualization were performed using Python 3.

Purpose and patterns

The purpose of this model is to investigate individual energetic dynamics and their link to animal community structure. In this study, we use the model to analyze energetics and coexistence in landscapes consisting of differently fragmented habitat. Coexistence is measured as coviability, which is the time to critical diversity loss. Here, this is the point in time at which less than half of the original species are present. To explore this measure of biodiversity and the underlying reasons for differences in the coviability, the model scales up from individual movement in home ranges of ten generic mammal species to community persistence across the landscape, that evolves from a successful home range establishment and reproduction. We initialize the community following the patterns of community composition with most individuals belonging to the smallest species and fewer individuals belonging to larger species. Additionally, we use the patterns of scaling of individual fat storage, home range size, field metabolic rate, and food intake with body mass for model evaluation. Finally, in this study, we aim to use the model to identify landscape compositions that are most beneficial to biodiversity.

Entities, State variables and Scales

The entities included in the model are landscape cells and mammal individuals. The landscape cells have a size of 10 m x 10 m, and the landscape consists of 100 x 100 cells which are arranged as a torus with periodic boundaries to avoid edge effects. The cells have the state variables location, habitat type, and resource amount (Table S2.1). The model distinguishes between two habitat types, as the cells can be hostile matrix providing no resources or suitable habitat for home range core cells and feeding.
The second entity, the mammal individuals, are considered non-volant, herbivorous, central-place foragers who frequently visit their home range core cell (Orians, 1979) and belong to ten different species characterized by their mean body mass. As body mass determines many physiological and ecological traits (Shingleton, 2010), the model uses this to assign other traits to the individuals based on allometric relations well established in the literature (Table S2.3). Several physiological traits are defined by allometries, such as basal metabolic rate, locomotion costs per distance, and maximum storage capacity. The model simulates individual energy dynamics by allowing individuals to ingest energy via foraging, which they then allocate to basal metabolic costs, digestion, locomotion costs, growth costs, pregnancy and lactation costs, and energy storage (following Sibly et al., 2013). Thus, individuals survive, grow and reproduce depending on their energy intake. Life history traits such as average lifespan of individuals, mean gestating and lactating periods, and litter size are also defined allometrically (Hamilton et al., 2011). In addition to energy level, body mass, and corresponding traits, age, sex, and home range core cell position are state variables of the individuals (Table S2.2). Furthermore, females have a reproductive state and count the duration of their pregnancy and lactation. The model has an additional set of non-allometric parameters (Table S2.4), for which we performed a thorough sensitivity analysis (Section 7).

Table S2.1: State variables of landscape cells

State variable	Unit	Description
Location	-	x and y coordinate
Habitat type	-	hostile matrix or suitable habitat
Resource amount	$g/day/100m^2$	available fodder biomass per cell

Table S2.2: State variables of the individuals

State variable	Unit	Description
Species	-	Species number (1 - 10) characterized by body mass
Expected body mass for species	*kg*	Individual body mass expected as adult
Actual body mass	*kg*	Current individual body mass dependent on growth status
Age	*days*	Age counter
Sex	-	Male or female (initial 1:1 ratio)
Core cell location	-	x and y coordinate of home range core cell
Energy level	g	Current level of energy stored and available (in g food)
Mother status	-	Females can be independent (without young), pregnant, or lactating
Mother counter	*days*	Duration of current pregnancy / lactation

The model proceeds in daily discrete timesteps and usually runs for 10 years.

Process overview and scheduling

In each daily timestep, the landscape resources regenerate, the individuals age by one day, they feed in their home range to ingest energy, and they spend this energy on basal costs, digestion, locomotion, and potentially on growing and serving offspring. At a certain age, the offspring become independent and search for their own home range. All individuals suffer from mortality due to fodder shortages, age, and additional background mortality (see Figure S2.1).

Design concepts

Basic principles: The model is based on former versions (Buchmann et al., 2011; Rohwäder & Jeltsch, 2022; Szangolies et al., 2022; Teckentrup et al., 2018). It is an individual-based dynamic spatial home range community model based on allometric relations and central place foraging. In comparison to the latest versions, the model bases its dynamics now on the metabolic processes of the individuals following principles proposed by Sibly et al. (2013).
Emergence: The mammal community structure emerges from individuals finding a home range and balancing energy intake and expenditure in each timestep successfully while also reproducing. The feeding range in each timestep emerges from the available fodder resources and the competition among individuals. Further, the energetic movement costs emerge from the distance moved to find enough food.
Objectives: Individuals try to cover their energetic objectives within the smallest possible range following the area-minimizing-principle (Mitchell & Powell, 2004). However, the energy demand is high as individuals try to maximize their energy storage while staying within their maximum possible home range.
Sensing: In model initialization, individuals have 100 attempts without locomotion costs to form their home range, thus they somehow sense a sufficient home range instead of running around unsuccessfully. Similarly, juveniles have ten attempts to find their own home range without paying locomotion costs, assuming some sensing of suitable habitat. Individuals are always aware of their own energy storage that they aim to fill.
Interaction: There are no direct interactions, but individuals compete for resources, and, to a certain extent, they forage in an order that depends on their mass and the abundance of their species. Rare and heavy species are assumed to have a competitive advantage.
Stochasticity: Apart from the slight sorting dependent on body mass and density (see Submodel

Table S2.3: Allometric traits of individuals as a function of body mass (M in kg)

Variable	Unit	Allometric relationship	Author	Name
Movement				
Maximum home-range area	ha	$56.23 \cdot M^{0.91}$	Kelt and Van Vuren (2001)	A1
Maximum natal dispersal	km	$3.31 \cdot M^{0.65}$	Sutherland et al. (2000)	A2
Life history				
Average maximum lifespan	days	$1766.53 \cdot M^{0.21}$	Hamilton et al. (2011)	A3
Gestation period	days	$64.14 \cdot M^{0.24}$	Hamilton et al. (2011)	A4
Lactation period	days	$57.16 \cdot M^{0.22}$	Hamilton et al. (2011)	A5
Litter size	#	$2.24 \cdot M^{-0.13}$	Hamilton et al. (2011)	A6
Mass of a neonate	g	$47.86 \cdot M^{0.93}$	Hamilton et al. (2011)	A7
Mass at weaning	g	$295.12 \cdot M^{0.91}$	Hamilton et al. (2011)	A8
Energetics				
Food share	-	$\frac{M}{0.001}^{-0.25}$	Hamilton et al. (2011)	A9
Maintenance costs	g food	$25.6 \cdot M^{0.737}$	Savage et al. (2004)	A10
Incremental locomotion costs	J/m	$10.7 \cdot M^{0.684}$	Calder (1996), Taylor and Heglund (1982)	A11
Postural locomotion costs	J/s	$6.03 \cdot M^{0.697} - 2.963 \cdot M^{0.737}$	calculated from above	A12
Routine speed	m/s	$0.06259 \cdot M^{0.217} \cdot e^{-0.002 \cdot ln(M \cdot 1000)^2}$	Cloyed et al. (2021)	A13
Maximum storage capacity	g food	$s \cdot 294.8 \cdot M^{1.19}$	Lindstedt and Boyce (1985)	A14

Table S2.4: Non-allometric parameters

Parameter	Unit	Default value	Description and Source
Habitat cover	%	5	Percentage of landscape that is considered habitat and has resources
Minimum mass	kg	0.01	Body mass of the lightest species
Maximum mass	kg	0.1	Body mass of the heaviest species
Initialized individuals	-	1000	Number of individuals
Order percentage	%	20	Percentage of individuals that forages in order to their body mass and conspecific density
Attempts to find home range	-	100	Number of attempts initialized individuals have to find a suitable home-range core cell
Attempts juvenile search	-	10	Number of attempts juveniles have to find a suitable home-range core cell within their dispersal distance
Background mortality	%	0.0333	Background mortality per day per individual (predation, disease, ...)
Assimilation efficiency	%	50	Percentage of energy in food that becomes available to individual (Hendriks, 1999; Peters, 1983)
Digestion costs	%	10	Percentage of energy costs of digestion / Heat increment of feeding (see Hindle et al., 2003; Nespolo et al., 2003)
Synthesizing flesh costs	g food	1300	Costs to synthesize 1 kg flesh for growth and for juveniles (Moses et al., 2008; Peters, 1983)
Maturity mass	%	95	Mass percentage (of target mass) of female when it can start reproducing
Mass of energy storage	kg	$\frac{1}{3930}$	How stored energy increases body mass (Schmidt-Nielsen, 1997)
Movement factor	-	3	Factor to account for non-straight movement path (calculated from data)
Mean grassland productivity	g/cell/day	68.5	Mean biomass amount per cell each timestep (Whittaker, 1975)
Available resource fraction	%	40	Percentage of biomass available as resource
Storage factor s	-	3	Factor to account for larger storage capacity (derived with data, see below)

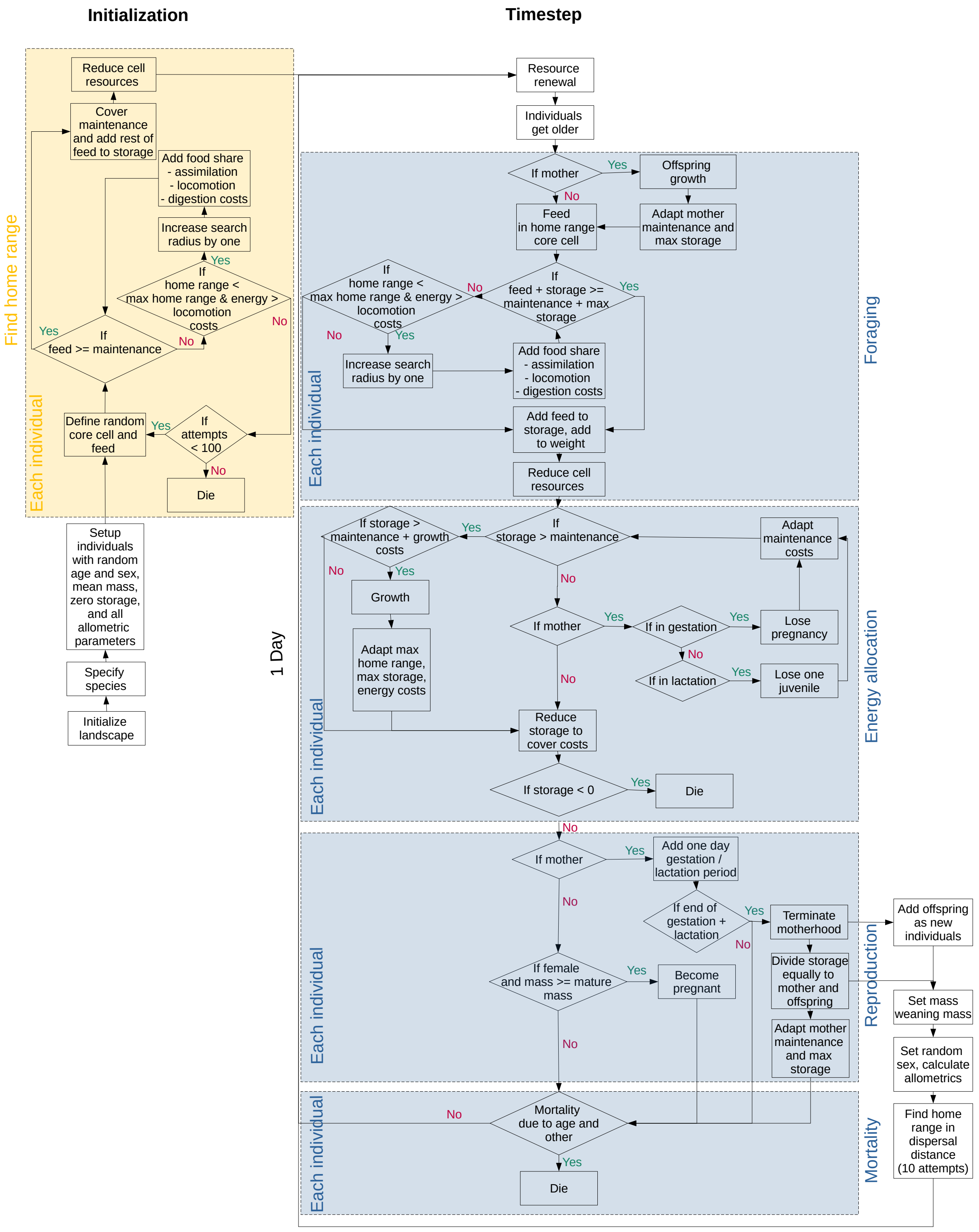

Figure S2.1: Flow Chart of model processes

section), the order in which individuals forage is random. Hence, individuals experience a different resource landscape each day. In the initialization, the location of the habitat patches in the landscape is stochastic, and the amount of resources provided by a patch is sampled from a normal distribution. Further, individuals body masses are also randomly drawn from a normal distribution around the mean of the species. Additional stochasticity is included as the initial 10 or 100 positions where individuals try to find a home range are chosen randomly from the habitat patches.
Observation: The main evaluation variable is the coviability, being the time when less than half of the initial species exist. Additionally, the community composition, i.e., population sizes over time are evaluated. On the individual level, the energy budget, movement radius, and number of successful offspring are returned by the model.
Input: The input to the model, defining the scenarios, is the fragmentation level. Further, the species that should be simulated can be defined. Optionally, all parameters from Table S2.4 can be adapted. In particular, habitat cover, maximum individual mass, initialized individuals, order percentage (how many individuals are sorted for foraging according to mass and density), attempts of adults and juveniles to find a home range, background mortality, maturity mass, and resource availability could not be specifically verified, or defined only the landscape scenario and thus could be adjusted as input of other model simulation scenarios.

Initialization

For landscape initialization, the total habitat cover and the level of fragmentation should be defined. The landscape is then initialized with random habitat locations according to the fragmentation level (see Figure S2.2). This landscape initialization algorithm works as follows. As long as not all habitat cells have been placed, the model runs through all cells again and again. If the current cell is already habitat and has non-habitat neighbor cells, one of these neighbors will become habitat. If the cell is not already habitat, there is a probability that it will become habitat. This probability depends on the initialized fragmentation level. If the probability is low, this will result in a low fragmented landscape, and if it is high, the landscape will be highly fragmented. Finally, when all habitat cells are placed, they get a resource level. The mean resource level per habitat patch is 27.4g, which is a percentage of the grassland productivity available as fodder for the individuals (see Buchmann et al., 2011; Whittaker, 1975).
In the initialization, the defined number of individuals (1000) is created first of all with their species. The species assignment and thus community structure follows a power-law distribution ($p(M) = K \cdot M^n$), with $p(M)$ being the probability of an individual to belong to species M (Buchmann et al., 2011). This power-law function results in most individuals being assigned to the smallest species, a few less to the next larger species, etc. (see Figure S2.2). Generally, the constant K is determined for the equation to integrate to one, and the exponent n defines the steepness of the body mass distribution among the species. In this model, n was set to -1.5, resulting in a realistic community structure (Buchmann et al., 2011).
After the assignment to a species, the specific body mass is drawn from the normally distributed body mass distribution, and all related trait values are calculated. The sex is defined randomly with a ratio of 1:1.
After this trait assignment, all introduced individuals try to establish a home range. This is done in the submodel Find home range. The individuals have a defined number of trials, where random habitat patches in the landscape are chosen at which they check whether the location is a suitable home range core cell. Therefore, individuals constantly increase the search radius by one until the maximum home range is reached, they can not cover the movement costs, or their energy demand is fulfilled. For this, in the initialization, it is already enough if individuals can cover their daily metabolic maintenance costs. If the demand is fulfilled, they define the cell as their home range core cell, if not, they go on checking cells or in the end, die. As all individuals are placed randomly and independently in the landscape, there is no territorial behavior included.

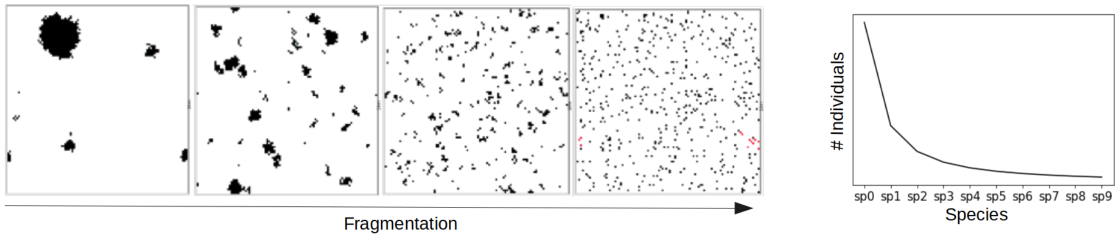

Figure S2.2: Initialization of landscapes composed of habitat patches in black that have different fragmentation levels from low to very high fragmentation (left) and initialization of the species community with individual abundances following a power-law distribution from small to large species (right).

Submodels

Update resources

Each day, all habitat cells redefine their amount of resources no matter if they have been foraged or not. The available resource amount is drawn from a normal distribution with a mean of 27.4g dry mass per day (percentage of grassland productivity; Whittaker, 1975) and a standard deviation of 0.7g dry mass per day, accounting for, e.g., weather differences.

The resource amount now depends on the day of the year. Resources follow a sinusoidal pattern to resemble simplified seasonality. The mean resource amount per day over the year remains the same (27.4g), but the summer maximum is 50% higher (41.1g) and the winter minimum is 50% lower (13.7). The curve of mean resources of a habitat cell per day of a year can be seen in Figure S2.3.

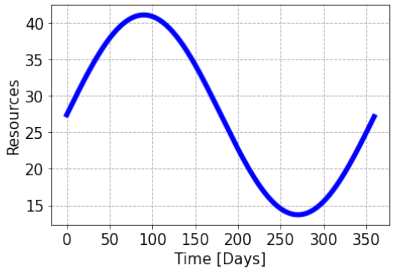

Figure S2.3: Pattern of resource availability per habitat cell over a year. Day 1 here is the beginning of spring.

Find home range

The find home range process is part of the initialization of a model run when newly created individuals have to find a home range. This home range of central place foragers is an area of a certain radius around a core cell that provides enough food resources to cover an individual's demand. There is a maximum allometric home range size for herbivorous mammals adapted from Kelt and Van Vuren (2001) (equation A1). When individuals search a home range they get assigned a randomly chosen habitat cell. First of all, they check whether in this one cell there already is enough food. If there is, they chose this as home range and feed there. If there is not, they increase the radius by one and check the adjacent cells (as depicted in Figure S2.4 by “1”). For this, they sum up the available fodder, which for each individual is a share of the total available resources on a cell (equation A9) minus assimilation, digestion, and locomotion costs. If individuals have enough energy, they stop searching, define this as their home range, and deplete the resources. During initialization, it is sufficient if they cover their maintenance, locomotion, and digestion costs. Individuals may forage more than they need as they try to fill their entire energy storage and they always feed on all cells within a certain radius. If an individual did not get enough food yet, it increases its radius by 1 again and again (as depicted in Figure S2.4 by “2” and “3”, etc.) until the maximum home range radius is reached, or the movement costs for the distances can not

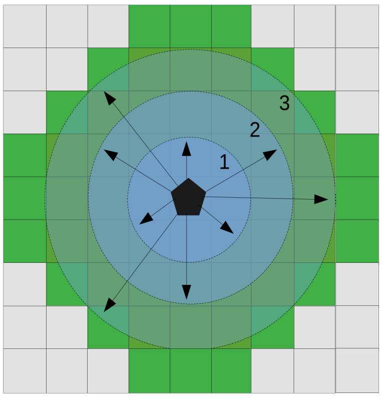

Figure S2.4: Home range search procedure

be covered. If an individual could not cover its energy demand at that location, it is assigned to another random habitat cell and tests this again. Each individual has a maximum of 100 trials to find a suitable home range but stops searching as soon as it found one. Food is depleted only if a suitable home range is found.

Check home range

The individuals have to feed each day. For this, they check the surroundings around their home range core cell again and forage until they got enough food. This foraging generally follows the procedure of home range search (Figure S2.4) but with the home range core cell already defined. The home range size can be adapted according to the current internal and landscape status but again can not exceed the allometric maximum home range size (equation A1). Individuals change their energy needs dependent on their body mass, their storage, and their reproductive stage, i.e., reproducing females have a higher energy demand. Further, due to competing individuals, the resource distribution around an individual may change. The order in which individuals forage is largely random, but individuals are slightly influenced by their weight and the density of conspecifics in the surroundings. More specifically, first, a new random order is determined each day by drawing a random number between zero and the maximum individual mass from a uniform distribution for each individual. Afterward, for randomly selected 20% of the individuals, this number is replaced by the product of the individuals' mass and a density factor. This density factor is the number of conspecifics within the maximum home range radius divided by the total number of individuals in that area. This slight sorting of only 20% of the individuals should symbolize the competitive advantage of heavier individuals and a density dependence on a very local scale. Due to the generally largely random sorting, individuals will experience different resource availability each day. Hence, home range size likely changes every timestep. During daily foraging, individuals continue searching for food until they either cover their entire daily needs and energy storage potential, they reach their maximum home range size, or they can not cover the locomotion costs to go to further away habitat cells. When individuals covered their needs, they stop searching. If they can not cover their maintenance costs with their energy intake and storage from former days, they will starve.

Find home range offspring

A special case of home range formation is the home range search of juveniles. This always happens after a successful reproduction period. Juveniles do not search the whole landscape but only within a certain allometric juvenile dispersal radius around the mother (equation A2, Sutherland et al., 2000). Within this radius, starting from the mother's home range core cell, juveniles have ten attempts to find and establish a suitable home range, following the same process as described above. Juveniles check whether a randomly selected habitat cell within the juvenile dispersal

distance is a suitable home range core cell. If a potential home range meets basal needs, the new individual will define this as its home range core cell and will forage there from then on. If the new individual does not find a suitable location within its possible range and trials, it will die or emigrate from the simulated area.

Energy maintenance, digestion and storage

Individuals acquire energy as they forage. The assimilation efficiency defines how much of that energy actually becomes available to the individuals. As we simulate herbivorous mammals, we assume a constant assimilation efficiency of 50% (Hendriks, 1999; Peters, 1983). Individuals try to meet their basal maintenance costs and fill their storage as much as possible. Both, basal requirements and maximum storage depend on the current body weight and can change over time (equations A10 and A14). For maximum storage, we have multiplied the allometrically defined value by a factor ($s = 3$) to obtain realistic fat storage values (see Section 6). Additional to maintenance, costs that always occur are digestion and locomotion costs. Both are applied directly during feeding, so individuals will try to fill their storage in addition to these costs. The digestion costs are also called the heat increment of feeding and are lost directly during the feeding process. These costs do not depend on body mass, but we just assume that they account for 10% of the total energy intake before assimilation (see Hindle et al., 2003; Nespolo et al., 2003). When energy is left after maintenance, digestion, and locomotion costs are covered, individuals invest it into growth and reproduction. If afterward, there is still some energy left, it remains stored and can be used in future days. However, this storage increases body mass, which increases the costs of locomotion on a daily basis (0.00025 kg/stored g food, see Schmidt-Nielsen, 1997).

Locomotion costs

To calculate the cost of movement, we use the distance traveled and theoretically the speed. In the model, individuals move from one cell to another in a straight line, which does not entirely resemble reality. Using tracking data from bank voles and striped field mice (Schirmer et al., 2019), we estimated a multiplication factor to compensate for extra movement and detours (see Section 3). According to this approach, each distance is multiplied by three. Furthermore, individuals always move back and forth from their home range core cell to each feeding cell, as they are central place foragers, so the distance is again doubled. For speed, we simply assume an allometric mean speed (equation A13, Cloyed et al., 2021). With distance and speed, activity time can be reconstructed. This activity time is needed to calculate the postural costs of movement that are associated with maintaining an upright posture (using equation A12). These are often overlooked but can account for a significant proportion of the energy associated with activity (Halsey, 2016). In addition to these postural costs, there are incremental costs that are directly calculated per distance (equation A11, Taylor and Heglund, 1982). These costs cover the energy required to actually change the location. They would vary with speed, but for simplicity, we use the average costs per distance.

Growth

Individuals already start growing when they are embryos and not independent individuals yet. When females get pregnant, their juveniles are initialized with an embryo mass, and from then on they grow each day. For embryos, a first growth curve applies that is based on Gompertz functions (Ricklefs, 2010). This curve defines how much they grow each day and how much energy females have to provide for this. After the defined gestation period, juveniles continue to grow following another growth curve for individuals (based on von Bertalanffy functions, see derivation in Sibly et al., 2013). Figure S2.5 shows the usual growing process for all species. Until weaning, juveniles are not independent individuals, and all young that belong to one litter grow in the same way only depending on the mothers weight. After weaning, individuals are initialized with their mass at weaning and from then on grow individually dependent on a growth curve again, but also dependent on their available energy. Contrary to when they still belong to their mother, adult individuals only grow if they have enough energy left for it after maintaining all other processes that need energy. If there is not enough energy left, individuals will not grow according to the growth curve on a given day.

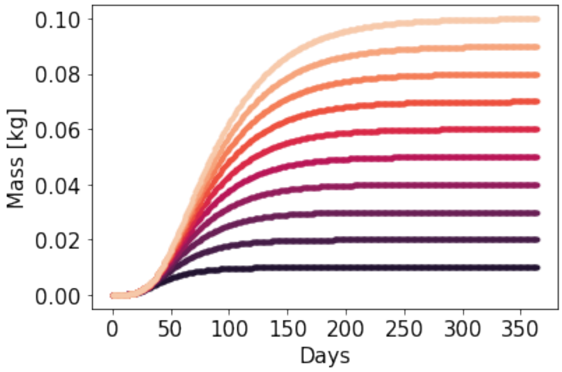

Figure S2.5: Individual growth following growth equations (Ricklefs, 2010; Sibly et al., 2013) starting from conception. Each color is a species.

Reproduction

Females that have reached a certain mass of maturity (95% of the expected final mass) become pregnant. Pregnant females measure the time of their pregnancy and lactation. They can only sustain their pregnancy if they can cover their increased maintenance costs due to the higher mass plus costs for synthesizing the flesh of the offspring (1300g food/kg flesh, Moses et al., 2008). Over pregnancy, the juveniles grow and increase the mothers energy demand. If females can not cover these costs they will lose the pregnancy. If they are already in the lactation phase, they will lose one offspring after another and may be able to serve a subset of them. When the allometrically defined gestation and lactation phase are over (equations A4 and A5, Hamilton et al., 2011), they stop being pregnant and then can get pregnant again in the next timestep. The litter size is allometrically defined dependent on the mother's mass (equation A6, Hamilton et al., 2011), and after lactation, these juveniles search their own home range. Juveniles inherit their mother's species and get assigned their body mass when leaving their mothers den (equation A8) with all the respective traits. The stored energy of the mother is equally divided between her and her offspring, as we assume that juveniles can already store energy during lactation if they are served well by their mother.

Mortality

All individuals face a certain mortality at the end of each day. Different reasons for mortality exist. The first reason can be a food shortage. If individuals do not have enough energy to cover maintenance costs, also not in their storage, they will die. There further is a mortality caused by age, with individuals dying when they are older than a random number from a normal distribution with an allometric mean maximum age for the species (equation A3, Hamilton et al., 2011) and 10% standard deviation of the average. Further, there is a background mortality due to predation, infection, etc., which is a 1% probability to die per month (0.0333% per day). Dead individuals are excluded from the model simulation.

3 Data evaluation

This TRACE element provides supporting information on: The quality and sources of numerical and qualitative data used to parameterize the model, both directly and inversely via calibration, and of the observed patterns that were used to design the overall model structure. This critical evaluation will allow model users to assess the scope and the uncertainty of the data and knowledge on which the model is based (Grimm et al., 2014).

Summary: There are a total of 14 allometric relations in the model plus three growth equations, that are all obtained from literature sources. Additionally, there are 17 non-allometric parameters in the model. Of those, some were again found in the literature. Some other parameters belong to model initialization such as habitat cover, simulated species mass, and number of initial individuals, and are simply defined based on the scenario. For one parameter, field data from another study was analyzed (movement factor). Some additional parameters could only be based on assumptions and some logic tests (order percentage, background mortality, maturity mass) or were rather technical and model specific without an equivalence in reality (attempts to find a home range). One parameter was adjusted using a pattern-oriented modelling approach (storage factor).

All the parameters belonging to the allometric relations were directly taken from the literature. The different literature sources used a variety of data to come up with these equations. Allometric equations and literature sources for them are given in Table 3. Growth equations are also visualized above in Section 2. Section 2 provides some detail on the assumptions for some parameters. Here, we provide detail on the estimation of the movement costs, as one central part of the model that was parameterized using field data. The derivation of the storage factor can be found below in Section 6.

Movement Distance

As energetic movement costs are one focus of our study, it was important to ensure realistic movement distances, not only realistic home range radii. In the model, individuals move from one cell to the next in a straight line. However, in nature, individuals would probably not move 10m in a straight line. To ensure realistic movement distances we used telemetry data from studies with bank vole and striped field mouse, which fall into the body mass range of our simulated species. The data was provided by Annika Schirmer, PhD (A) and Sophie Eden (B) (partly published in Schirmer et al., 2019). The two different studies had different resolutions (data points every 20 min or every 6 min) and we evaluated the data separately. Our aim was to establish a factor by which we can multiply the direct distance to achieve a realistic movement distance. We used two different approaches to get such a factor from the field data.

First, we estimated how long an individual needed to walk 10m which is the resolution of the simulated landscape and then we calculated the fraction of real distance versus direct distance that was usually covered in such a time frame (see scheme in Figure S2.6). Although the time needed to walk 10m differed for the two data sets, this relation of real versus direct distance was fairly similar in both data sets (A: 3.03, B: 2.8).

In a second calculation, we estimated how far individuals usually moved within a 10 m x 10 m frame, which is the size of a simulated landscape cell (see Figure S2.7). In the model, this distance is a straight line, which would be between 10 m minimum and 14.14 m maximum (diagonal through a 10 m x 10 m square), resulting in a mean of 12.07 m. In the tracking data, we took the mean of all possible 10 m x 10 m windows and found distances of 36.03 m (A) and 35.2 m (B). The relation between those values and the mean model value is 2.99 (A) and 2.92 (B).

As in both estimation approaches and with both data sets of different resolutions, more or less similar values were estimated, we decided to use 3 as factor to multiply the direct distance. We rounded the value to 3 rather than taking the exact mean of all values, as the estimation method and data do not allow for very exact measurement and in our opinion, a whole number does represent this better than several decimals.

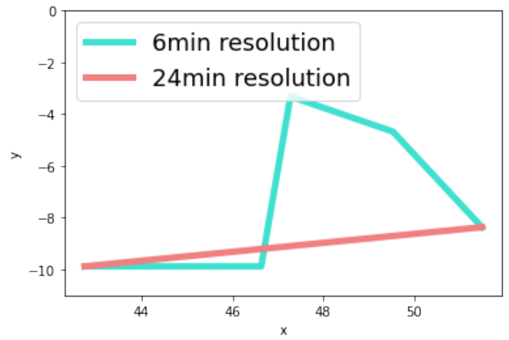

Figure S2.6: Movement distance estimation method 1

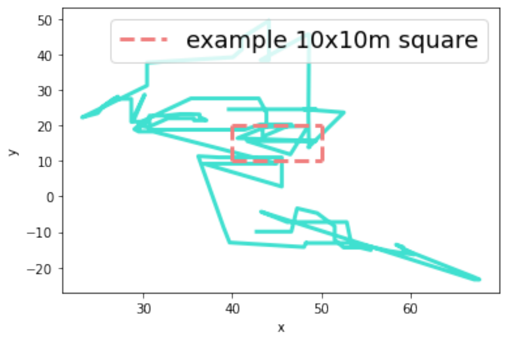

Figure S2.7: Movement distance estimation method 2

Movement Costs

Movement costs can be separated into incremental and postural costs (Halsey, 2016). Usually, only incremental costs are considered, for which we also found proper allometric relations (Calder, 1996; Taylor & Heglund, 1982). However, estimating postural costs is more challenging. As there is no direct calculation or allometry of postural costs, we derived it under some assumptions. We used total activity costs for terrestrial locomotion per second from Calder (1996), Taylor and Heglund (1982) and subtracted the basal metabolic costs (Savage et al., 2004) scaled per second and the incremental costs. This resulted in costs for posture per time. To get those costs per distance we divided the costs per time by the routine locomotion speed for walking (Cloyed et al., 2021). This calculation resulted in an estimation of postural locomotion costs per distance dependent on body mass. Although this is a rather rough estimation including the uncertainty of four other equations, we think considering such postural costs is important.

Incremental locomotion cost (Calder, 1996; Taylor & Heglund, 1982):

$$\$[J/m] = 10.7M^{0.684}$$

Total metabolic rate for terrestrial locomotion (Calder, 1996; Taylor & Heglund, 1982):

$$E[J/s] = 6.03M^{0.697} + 10.7M^{0.684}v$$

Basal metabolic rate (Savage et al., 2004):

$$E[kJ/day] = 256M^{0.737}$$
$$E[J/s] = 2.96M^{0.737}$$

$\rightarrow$ Postural costs per time :

$$Total - Incremental - Basal$$
$$E[J/s] = 6.03M^{0.697} - 2.96M^{0.737}$$

Routine locomotion speed (Cloyed et al., 2021):

$$Walk[log(m/s)] = 0.217M[log(g)] - 0.002M^2 - 4.27$$
$$Walk[m/s] = 0.06259M^{0.217}[kg] \cdot e^{-0.002ln(1000M)^2}$$

$\rightarrow$ Postural costs per distance :

$$\frac{Postural/Time}{Speed}$$
$$E[J/m] = \frac{6.03M^{0.697} - 2.96M^{0.737}}{0.06259M^{0.217}[kg] \cdot e^{-0.002ln(1000M)^2}}$$

4 Conceptual model evaluation

This TRACE element provides supporting information on: The simplifying assumptions underlying a model's design, both with regard to empirical knowledge and general, basic principles. This critical evaluation allows model users to understand that model design was not ad hoc but based on carefully scrutinized considerations (Grimm et al., 2014).

Summary: The metabolic community model expands a previous individual-based home-range community model without energetics (Buchmann et al., 2011; Rohwäder & Jeltsch, 2022; Szangolies et al., 2022). We discuss here the simplifying assumptions of the original home-range modelling and the new individual energetics.

There are some underlying simplifying assumptions for the different parts of the model. First of all, in the existing movement part of the model, all individuals are central-place foragers. This means that they always move back and forth from their home range core cell to each foraging cell. Central-place foraging is common, particularly for small mammals (Orians, 1979), but may not apply for all species when extrapolating the model to other species communities. Another strong simplification is that the home ranges of the individuals are circular. They are simply defined by a radius around the home range core cell that can change every day. The home range may be only theoretically circular, but the individual only moves to actual habitat cells that may just be in a corner of this radius, leading to practically non-circular home ranges. Nevertheless, theoretically, we only allow circular home ranges.

In the new energetic part of the model, there are also some assumptions. The most obvious one is that we follow the Sibly et al. (2013) approach for energy allocation. Animals take in energy by foraging. From that, they always have to cover maintenance, digestion, and locomotion costs. To simplify locomotion costs, we assume a constant mean speed. Reproducing females then invest in reproduction and only if energy is left do they grow. Non-reproducing individuals grow if they have enough energy left. We assume that there is no specific energy management strategy, but the energy needed for activity will just add to maintenance costs (see other strategies in, i.e., Careau et al., 2021). The energy intake depends on resource availability and foraging success. For foraging, individuals are sorted mostly random each day. Yet, we implemented a slight sorting that gives heavier and locally rare species a little advantage. This is based on the assumptions that heavy species might be competitively dominant and there can be a slight local density dependence. Yet, as these processes should not determine whole model outcome, we only gave this advantage to a certain percentage of individuals. We determined this percentage to be robust against slight changes. Another assumption is that individuals will always try to fill their storage completely when foraging. This might not always be optimal as it may lead to high movement costs for not as much energy intake. Yet, we use this as simplification as animals are most likely not always optimally informed about the landscape and will often try to feed as much as possible. Further, we validated that this leads to realistic fat storage and food intake (Sections 6 and 8).

In the way reproduction was implemented in the model, we assume that females will always get pregnant if they have reached a certain mass of maturity and are not already pregnant. The mass of maturity is already another assumption. Although it is reported that a certain mass is needed to reach maturity (i.e., Sibly et al., 2013), typically rather a certain maturity age is used (Hamilton et al., 2011). We just defined the mass of maturity at 95% of the expected mean final mass for the species. Once they reached this mass, females in the model will always be pregnant, which may not be true in reality. In the model, they may lose their pregnancy when they do not have enough energy, but then they may try to get pregnant again the next day. We do not consider males in the reproduction, we simply assume that there will be a male available for mating. Additionally, we assume that for each juvenile, females have to cover additional maintenance costs, but also have a larger storage. This is based on the assumption that each embryo and juvenile can store energy already when they are still with their mother. In the model, this has to be recorded by the mother as the young are not independent individuals yet. This is also why we split the energy storage of the mother at the end of the lactation period through her and her offspring.

These simplifying assumptions of the presented metabolic community model may be reconsidered in future model development and can be adapted if needed for specific research questions.

5 Implementation verification

This TRACE element provides supporting information on: (1) whether the computer code for implementing the model has been thoroughly tested for programming errors and (2) whether the implemented model performs as indicated by the model description (Grimm et al., 2014).

Summary: The computer code was continually tested during model development to ensure the functionality of each submodel. Visual inspection of submodel performance was done using the NetLogo graphical user interface (GUI), and the extension *profiler* was used for debugging purposes. Hence, we made sure that each submodel performed as indicated by this TRACE. Moreover, to really exclude programming issues, we implemented a similar model in a second programming language. The model was also programmed in Python 3, resulting in similar results.

We have thoroughly tested the model and all its submodels to ensure that it works as expected. Several model output parameters were validated as described in other sections of this TRACE. As the main novelty of this model version was the energetics, outputs related to this (e.g., field metabolic rate) were particularly evaluated and tracked throughout the simulation.
We used various options available in NetLogo to verify the functioning of the implementation, from print statements to plots and debugging using the extension *profiler*. The parameter ”debug” is still included in the program.
Additionally, we programmed a version of the model in another programming language. By implementing a similar model in Python and comparing the simulation output, we ensure that in neither of the implementations basic programming errors influence the output. The Python version of the model has less functionality, i.e., less output variables are implemented, and contains some assumptions to allow faster simulation, such as an aggregated home range search of offspring. Nonetheless, we could use the Python model to perform community simulations under fragmentation levels similar to the NetLogo simulation. We evaluated the coviability and found similar results as in the NetLogo simulation (compare Figure S2.8 and Figure S2.9). As the two versions were programmed independent of each other, but led to similar results, we conclude that the implementations work well and that results such as high coviability at medium fragmentation are robust.

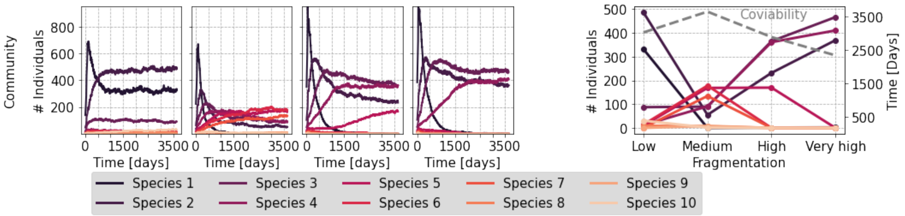

Figure S2.8: Simulations of population sizes over time performed by the Python model. Population sizes are the mean of 5 repetitions for each fragmentation scenario. On the right, the population sizes at the end of the simulation and the coviability are shown (grey dashed line and right axis).

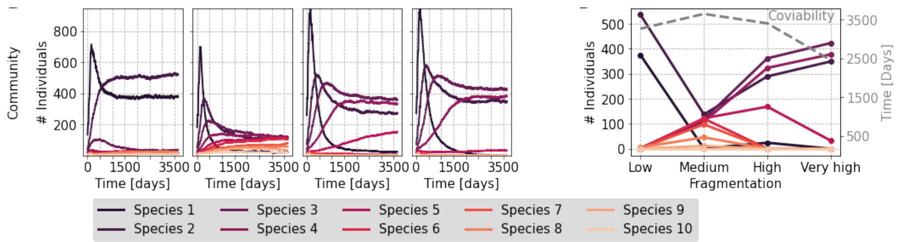

Figure S2.9: Simulations of population sizes over time performed by the NetLogo model. Population sizes are the mean of 20 repetitions for each fragmentation scenario. On the right, the population sizes at the end of the simulation and the coviability are shown (grey dashed line and right axis).

6 Model output verification

This TRACE element provides supporting information on: (1) how well model output matches observations and (2) how much calibration and effects of environmental drivers were involved in obtaining good fits of model output and data (Grimm et al., 2014).

Summary: We specifically derived one parameter from patterns found in literature. Other parameters were directly found in the literature or were not possible to calibrate due to missing related output patterns. We additionally verified how well general model output matches independent patterns (Section 8).

While allometric relations are common and well understood, and we took the associated parameters from the literature without further validation, there were also some uncertain parameters. Where possible, we based these parameter values on existing patterns.
There was an allometric relationship for energy storage (Lindstedt & Boyce, 1985), yet we had to adjust it for resulting individual storage to fit literature data of fat storage. Before adjustment, the allometric equation was not for maximum storage and resulting storage values in model simulation where to low. Hence. we applied a factor to the allometric relation so that the simulated fat storage fits literature data from diverse sources and for several species, all of which together gave a well-fitting linear regression of body mass and fat mass (Figure S2.10). To estimate the best fitting factor value, we used a low fragmented landscape, as field studies from which we obtain literature data are usually conducted in connected habitats. We simulated varying values for the parameter storage factor and evaluated the outcome patterns. The factor value 3 resulted in the fit shown in Figure S2.10. This parameter was the only one for which we specifically used pattern-oriented estimation.

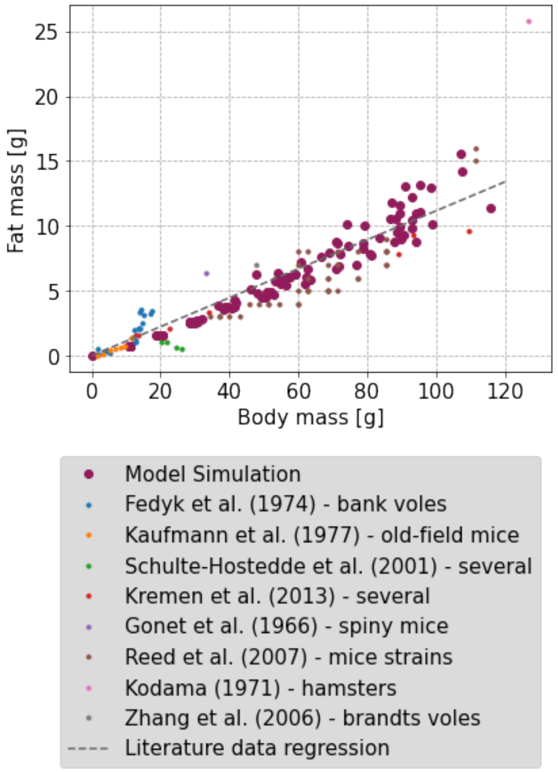

Figure S2.10: Fit of simulated energy storage with fat masses from literature data (Fedyk, 1974; Gonet et al., 1966; Kaufman & Kaufman, 1977; Kodama, 1971; Kremen et al., 2013; Reed et al., 2007; Schulte-Hostedde et al., 2001; Zhang & Wang, 2006)

7 Model analysis

This TRACE element provides supporting information on: (1) how sensitive model output is to changes in model parameters (sensitivity analysis), and (2) how well the emergence of model output has been understood (Grimm et al., 2014).

Summary: To ensure the robustness of model outcome, we performed a two-step sensitivity analysis similar to Ayllón et al. (2016) and Gallagher et al. (2021). This analysis included firstly an individual screening of all parameters (Morris, 1991) and secondly a global sensitivity analysis for the most sensitive parameters identified in the screening (Sobol, 2000). The output patterns coviability, population sizes, energy intakes, and field metabolic rates were evaluated.

We performed a sensitivity analysis for all non-allometric model parameters that were not taken directly from the literature (Table S2.4). This sensitivity analysis was structured in two steps (as in Ayllón et al., 2016; Gallagher et al., 2021). First, the 12 model parameters were screened by varying them over four levels with a change of up to 10% around the default value in the model following the Morris elementary effects method (Campolongo et al., 2007; Morris, 1991). For each parameter, 50 relative effects of changes in the value, called elementary effects, were estimated. This resulted in 650 model runs ($50 \cdot (12{+}1){=}650$) for this sensitivity step, that each run for the usual duration of 10 simulation years. From these simulations, we ranked the parameters according to their influence on some model outputs. As outputs, we considered the coviability, the mean population size per species, the mean daily energy intake per species, and the mean field metabolic rate per species. We evaluated the latter three per species, but ultimately took the average of the effect sizes of all species. This resulted in four evaluated outcomes. The means of the distributions of the elementary effects of each of these outputs were finally used as sensitivity indices and the percentage of maximum sensitivity was calculated (Table S2.5). For each output variable, the parameters with effect sizes greater than 90% were selected for a second step of sensitivity analysis. As some parameters were ranked high for more than one of the outputs, this resulted in six parameters for further investigation.
Coviability was most influenced by the habitat cover, the maximum individual mass (the mean mass of each species), the maturity mass, and the movement factor. The population sizes of the species were most influenced by the maturity mass and the number of initialized individuals. Energy intake and field metabolic rate are closely related and therefore most influenced by the same parameters. These are the habitat cover, the maximum individual mass, and the percentage of resources available to the individuals.
Therefore, we performed the second step of the sensitivity analysis with the six parameters habitat cover, maximum individual mass, number of initialized individuals, maturity mass, movement factor, and percentage of available resources. Here, we used a full variance decomposition technique (Saltelli et al., 2010; Sobol, 2000) to estimate the first-order and total-effect indices. The six parameters were varied similarly to the previous Morris analysis step and the same output variables were used. To assess the influence of the parameters on the model output variance, the parameters for the Sobol sensitivity analysis were randomly combined. A Monte Carlo sampling matrix was used, again with a size of 50. This resulted in 400 model runs ($50 \cdot (6{+}2){=}400$). A confidence level of 95% was computed in addition to the mean (Table S2.6 and S2.7). This analysis was performed using the SALib package in Python (Herman and Usher 2017).
The results indicate, that the individual influence (first order indices) of all analyzed parameters was at least below 40% (Table S2.6). The reported effect sizes for population size, energy intake, and field metabolic rate are means of the species values, and the actual effect sizes for each species may deviate from this. Hence, the reported mean values give an impression of the influence, but have to be interpreted with caution. In addition to the individual influence, contributions of interactions between parameters to the model output variance are identified by estimating the influence of parameters with included variance caused by their interactions with any or all of the other input parameters (Table S2.7). These resulting total-effect indices show a much stronger influence on model output variance than the first-order indices indicating combined effects of parameters and the high complexity of model dynamics. Coviability was most strongly influenced by the habitat

cover and the movement factor. Individual population sizes, energy intake, and field metabolic rate were influenced by all parameters more or less similarly and strongly, which may be due to strong correlative effects of all parameters on each other. There are also rather large confidence intervals likely resulting from the variable interactions between parameters.
Generally, the parameters identified as strongly influential need to be implemented with caution. Parameters connected to the landscape, like the number of the total habitat cover and the available resource percentage affect the coviability and the community composition rather strongly as they alter species competition advantages by changing available habitat and movement needs. Such landscape information can, for example, be achieved for specific landscapes by aerial imaging, but sometimes field studies are also necessary. Rather individual related parameters, like the maturity mass and movement factor relate to reproduction success and movement costs. Hence, they can more or less directly affect energetics and fitness of individuals, which is probably why they have such a strong effect. For these variables, more field observations and tracking of mammal communities would be helpful to provide more background for their definition. The number of initialized individuals and maximum simulated mass are more related to model initialization and could only be inspired by field measures of current situations, but may be adapted dependent on the simulated system.

Table S2.5: Morris Sensitivity Analysis

Parameter	Coviability	Population size	Energy intake	Field metabolic rate
Habitat Cover	**98.6**	86.47	**90.09**	**92.16**
Maximum mass	**100.0**	80.99	**99.50**	**100.0**
Initialized individuals	88.6	**91.19**	86.0	86.29
Order percentage	71.8	80.51	67.81	68.15
Attempts to find home range	70.4	76.74	63.79	64.75
Attempts juvenile search	76.8	75.53	70.73	69.5
Background mortality	79.7	77.21	77.38	76.49
Maturity mass	**99.1**	**92.40**	73.73	72.96
Movement factor	**95.0**	85.10	87.84	88.86
Available resource fraction	87.4	85.91	**93.29**	**93.62**
Resource variability	77.1	76.37	84.53	85.28
Storage factor	85.3	88.12	80.96	82.17

Table S2.6: Sobol sensitivity analysis absolut first order indices (for population size, energy intake, and field metabolic rate it is the mean over all species specific values)

Parameter	Coviability	Population size	Energy intake	Field metabolic rate
Habitat Cover	0.238 +/- 0.381	0.179 +/- 0.363	0.301 +/- 0.371	0.251 +/- 0.387
Maximum mass	-0.028 +/- 0.356	0.090 +/- 0.323	0.235 +/- 0.376	0.202 +/- 0.402
Initialized individuals	-0.062 +/- 0.335	0.145 +/- 0.371	0.236 +/- 0.385	0.122 +/- 0.410
Maturity mass	0.063 +/- 0.313	0.200 +/- 0.501	0.307 +/- 0.378	0.289 +/- 0.356
Movement factor	0.017 +/- 0.419	0.167 +/- 0.422	0.242 +/- 0.439	0.298 +/- 0.351
Available resource fraction	0.089 +/- 0.354	0.115 +/- 0.398	0.371 +/- 407	0.258 +/- 0.357

Table S2.7: Sobol sensitivity analysis absolut total-effect-indices (for population size, energy intake, and field metabolic rate it is the mean over all species specific values)

Parameter	Coviability	Population size	Energy intake	Field metabolic rate
Habitat Cover	1.081 +/- 0.37	1.114 +/- 0.663	1.085 +/- 0.476	1.145 +/- 0.493
Maximum mass	0.833 +/- 0.307	0.926 +/- 0.501	0.922 +/- 0.403	1.075 +/- 0.387
Initialized individuals	0.756 +/- 0.297	1.016 +/- 0.550	1.042 +/- 0.443	1.045 +/- 0.444
Maturity mass	0.820 +/- 0.353	1.247 +/- 0.688	1.104 +/- 0.458	1.118 +/- 0.451
Movement factor	1.034 +/- 0.365	1.200 +/- 0.686	1.081 +/- 0.406	1.047 +/- 0.394
Available resource fraction	0.724 +/- 0.291	1.239 +/- 0.774	0.962 +/- 0.404	0.920 +/- 0.373

Comparing our two model versions, one with static resources over the year and one with a seasonal resource pattern, we see that population sizes do follow the resource availability, but relative species abundance in a community and overall species richness are not strongly influenced by these differences in resources (Figure S2.11). Similarly, the intensity of the seasonality does not change these patterns, at least within tested limits. Consequently, our model is relatively robust against moderate seasonal changes in resource level.

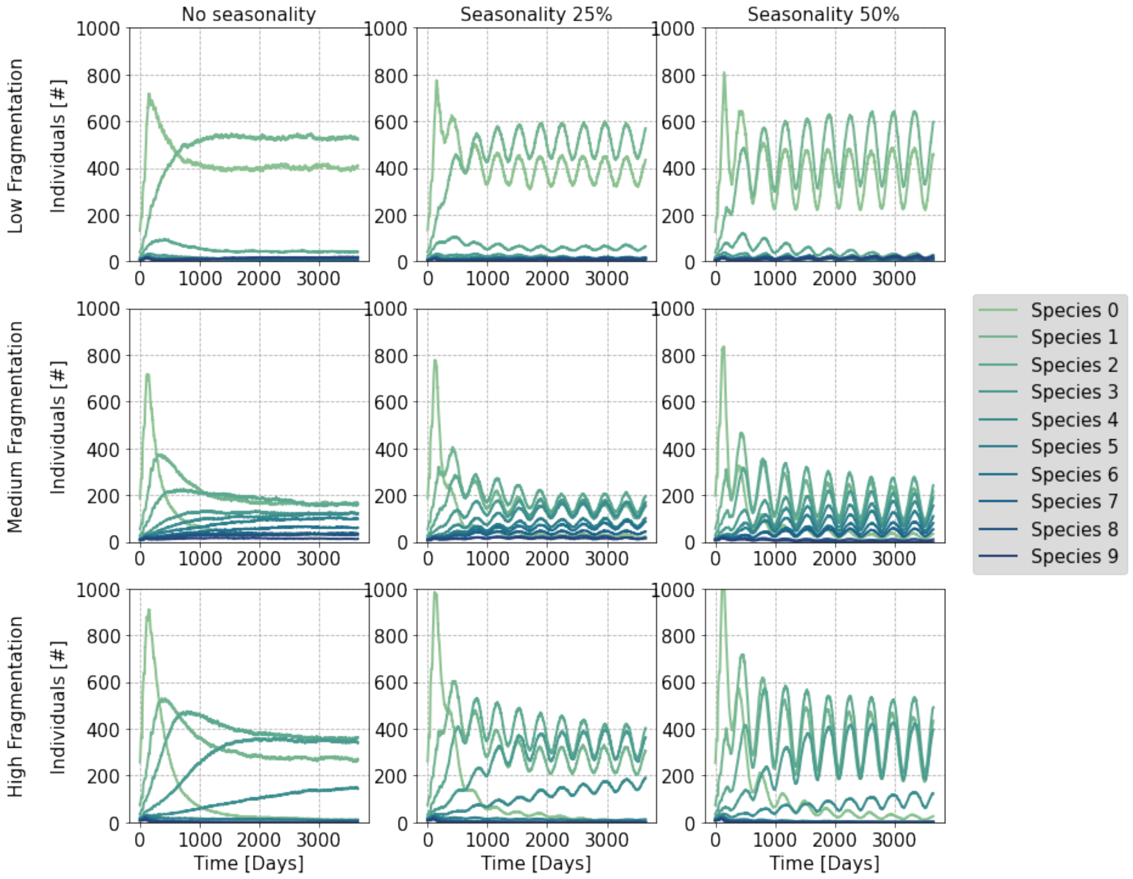

Figure S2.11: Population dynamics in a community of species in differently fragmented landscapes and with different seasonal patterns of resources. Szangolies et al. (2024) uses no seasonality and the current study uses 50% seasonality, meaning summer and winter deviations from the mean resource level by 50% of this mean. Additionally we show 25% seasonality with summer and winter deviations of 25% of mean resource level.

8 Model output corrobation

This TRACE element provides supporting information on: How model predictions compare to independent data and patterns that were not used, and preferably not even known, while the model was developed, parameterized, and verified. By documenting model output corroboration, model users learn about evidence, which, in addition to model output verification, indicates that the model is structurally realistic so that its predictions can be trusted to some degree (Grimm et al., 2014).

Summary: The two main processes of the model are foraging in home ranges and individual energetic dynamics. We aimed to validate the outcome of both of these processes. Hence, we compared the scaling of emergent home range sizes and metabolic rates to independent literature data. The energetic variables field metabolic rate and energy intake of individuals in different reproductive states were validated against independent field data. We found nice fits with literature regressions, which increase trust in our model and simulation results.

Earlier non-energetic versions of the model have already been tested and have proven their usefulness (Buchmann et al., 2011; Rohwäder & Jeltsch, 2022; Szangolies et al., 2022). The main novelty now is the energetics. Yet, this influences the entire model dynamics. Hence, we validated again, apart from energetic output, also the home range sizes. This was done by running model simulations with a community in a low fragmented landscape, as field studies are usually conducted in contiguous habitats. The average outputs of ten model simulations were analyzed.
While home range sizes depend on resource availability per habitat cell (which is a percentage of the grassland productivity given by Whittaker, 1975), especially the scaling with mass is largely based on the competition dynamics among individuals as well. The scaling of the simulated home range sizes with body mass matched well with database data and literature regressions (see Figure S2.12; Jones et al., 2009; Kelt and Van Vuren, 2001; Tucker et al., 2014). Variability is present in both, simulated and literature data, but the range matches well.
To further assess whether the energetics in the model work appropriately, we compared the emerging field metabolic rate and the food intake from the model with independent field data. Energy costs can be greatly increased by reproduction, so we separated this analysis for reproducing females and non-reproducing males (males have no cost for reproduction). Figure S2.12 shows the good fit of the model to the literature data. As also described in the main manuscript, there is some discrepancy between literature sources regarding the field metabolic rate allometry (Nagy, 2005; Speakman, 1999), but the simulated data are in range of field data (Nagy et al., 1999) and show similar scaling with body mass. For very small species, the energy intake in the model is slightly higher than expected from the data, and for reproducing females of larger species it is slightly lower than expected. Nonetheless, considering the individual data points from the literature source (Speakman, 2008), the simulated values are still within a realistic range (Figure S2.12).
Further verification of realism would require more field data on wild species communities. For example, to date there are no simultaneous energy measurements of individuals living together in a community in the wild. There are also no comparisons in the literature of these energetics as influenced by different landscapes. Such measurements would be challenging in the wild, which is also why a simulation model is very useful. In the absence of such data, we can still conclude that the model works as expected and that the results match some of the patterns available in the literature.

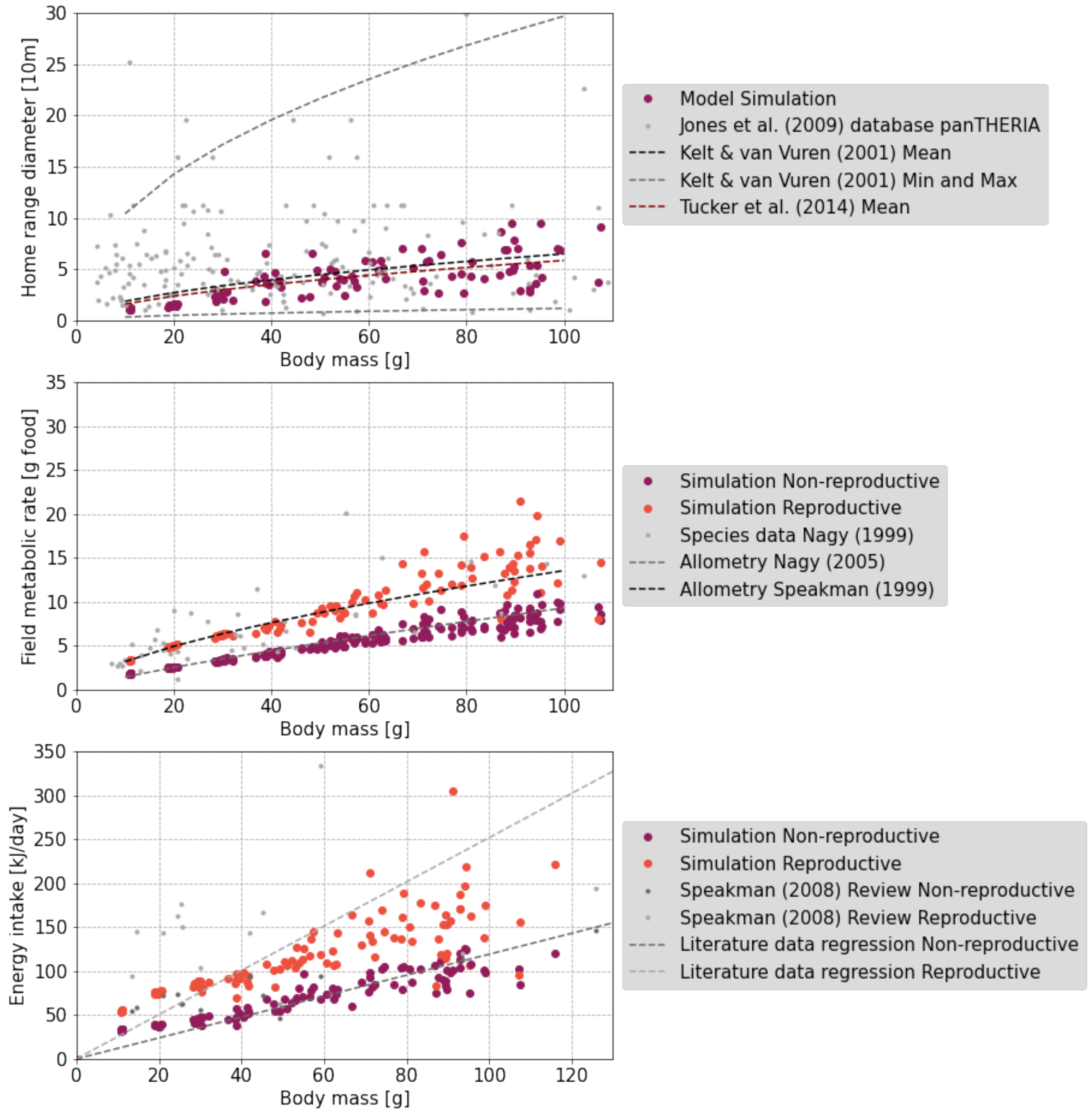

Figure S2.12: Validation fit of emerging home range size, field metabolic rate, and food intake in model simulations compared to literature data and regressions (which accumulated data for several species)

References

Ayllón, D., Railsback, S. F., Vincenzi, S., Groeneveld, J., Almodóvar, A., & Grimm, V. (2016). InSTREAM-Gen: Modelling eco-evolutionary dynamics of trout populations under anthropogenic environmental change. *Ecological Modelling*, *326*, 36–53. https://doi.org/10.1016/j.ecolmodel.2015.07.026

Brandl, S. J., Lefcheck, J. S., Bates, A. E., Rasher, D. B., & Norin, T. (2023). Can metabolic traits explain animal community assembly and functioning? *Biological Reviews*, *98(1)*, 1–18.

Buchmann, C. M., Schurr, F. M., Nathan, R., & Jeltsch, F. (2011). An allometric model of home range formation explains the structuring of animal communities exploiting heterogeneous resources. *Oikos*, *120(1)*, 106–118.

Calder, W. A. (1996). *Size, function, and life history*. Courier Corporation.

Campolongo, F., Cariboni, J., & Saltelli, A. (2007). An effective screening design for sensitivity analysis of large models. *Environmental modelling & software*, *22(10)*, 1509–1518.

Careau, V., Halsey, L. G., Pontzer, H., Ainslie, P. N., Andersen, L. F., Anderson, L. J., Arab, L., Baddou, I., Bedu-Addo, K., Blaak, E. E., Et al. (2021). Energy compensation and adiposity in humans. *Current Biology*, *31(20)*, 4659–4666.

Cloyed, C. S., Grady, J. M., Savage, V. M., Uyeda, J. C., & Dell, A. I. (2021). The allometry of locomotion. *Ecology*, *102(7)*, e03369.

Fedyk, A. (1974). Gross body composition in postnatal development of the bank vole. i. growth under laboratory conditions. *Acta Theriologica*, *19(26)*, 381–401.

Gallagher, C. A., Grimm, V., Kyhn, L. A., Kinze, C. C., & Nabe-Nielsen, J. (2021). Movement and seasonal energetics mediate vulnerability to disturbance in marine mammal populations. *The American Naturalist*, *197(3)*, 296–311.

Gonet, A. E., Stauffacher, W., Pictet, R., & Renold, A. E. (1966). Obesity and diabetes mellitus with striking congenital hyperplasia of the islets of langerhans in spiny mice (acomys cahirinus) i. histological findings and preliminary metabolic observations. *Diabetologia*, *1(3-4)*, 162–171.

Grimm, V., Augusiak, J., Focks, A., Frank, B. M., Gabsi, F., Johnston, A. S., Liu, C., Martin, B. T., Meli, M., Radchuk, V., Et al. (2014). Towards better modelling and decision support: Documenting model development, testing, and analysis using trace. *Ecological modelling*, *280*, 129–139.

Grimm, V., Railsback, S. F., Vincenot, C. E., Berger, U., Gallagher, C., DeAngelis, D. L., Edmonds, B., Ge, J., Giske, J., Groeneveld, J., Et al. (2020). The odd protocol for describing agent-based and other simulation models: A second update to improve clarity, replication, and structural realism. *Journal of Artificial Societies and Social Simulation*, *23(2)*.

Halsey, L. G. (2016). Terrestrial movement energetics: Current knowledge and its application to the optimising animal. *Journal of Experimental Biology*, *219(10)*, 1424–1431.

Hamilton, M. J., Davidson, A. D., Sibly, R. M., & Brown, J. H. (2011). Universal scaling of production rates across mammalian lineages. *Proceedings of the Royal Society B: Biological Sciences*, *278(1705)*, 560–566.

Hendriks, A. J. (1999). Allometric scaling of rate, age and density parameters in ecological models. *Oikos*, 293–310.

Hindle, A. G., McIntyre, I. W., Campbell, K. L., & MacArthur, R. A. (2003). The heat increment of feeding and its thermoregulatory implications in the short-tailed shrew (blarina brevicauda). *Canadian journal of zoology*, *81(8)*, 1445–1453.

Jones, K. E., Bielby, J., Cardillo, M., Fritz, S. A., O'Dell, J., Orme, C. D. L., Safi, K., Sechrest, W., Boakes, E. H., Carbone, C., Et al. (2009). Pantheria: A species-level database of life history, ecology, and geography of extant and recently extinct mammals: Ecological archives e090-184. *Ecology*, *90(9)*, 2648–2648.

Kaufman, G. A., & Kaufman, D. W. (1977). Body composition of the old-field mouse (peromyscus polionotus). *Journal of Mammalogy*, *58(3)*, 429–434.

Kelt, D. A., & Van Vuren, D. H. (2001). The ecology and macroecology of mammalian home range area. *The American Naturalist*, *157(6)*, 637–645.

Kodama, A. (1971). In vivo and in vitro determinations of body fat and body water in the hamster. *Journal of applied physiology*, *31(2)*, 218–222.

Kremen, N., Calvert, C., Larsen, J., Baldwin, R., Hahn, T., & Fascetti, A. (2013). Body composition and amino acid concentrations of select birds and mammals consumed by cats in northern and central california. *Journal of Animal Science*, *91(3)*, 1270–1276.

Lindstedt, S. L., & Boyce, M. S. (1985). Seasonality, fasting endurance, and body size in mammals. *The American Naturalist*, *125(6)*, 873–878.

Mitchell, M. S., & Powell, R. A. (2004). A mechanistic home range model for optimal use of spatially distributed resources. *Ecological Modelling*, *177(1-2)*, 209–232.

Morris, M. D. (1991). Factorial Sampling Plans for Preliminary Computational Experiments. *Technometrics*, *33(2)*, 161–174. https://doi.org/10.1080/00401706.1991.10484804

Moses, M. E., Hou, C., Woodruff, W. H., West, G. B., Nekola, J. C., Zuo, W., & Brown, J. H. (2008). Revisiting a model of ontogenetic growth: Estimating model parameters from theory and data. *The American Naturalist*, *171(5)*, 632–645.

Nagy, K. A. (2005). Field metabolic rate and body size. *Journal of experimental biology*, *208(9)*, 1621–1625.

Nagy, K. A., Girard, I. A., & Brown, T. K. (1999). Energetics of free-ranging mammals, reptiles, and birds. *Annual review of nutrition*, *19(1)*, 247–277.

Nespolo, R. F., Bacigalupe, L. D., & Bozinovic, F. (2003). The influence of heat increment of feeding on basal metabolic rate in phyllotis darwini (muridae). *Comparative Biochemistry and Physiology Part A: Molecular & Integrative Physiology*, *134(1)*, 139–145.

Orians, G. (1979). On the theory of central place foraging. *Analysis of ecological systems*, 157–177.

Peters, P. (1983). *Ecological implication of body size*. Cambridge, Mass.: Harvard Univ. Press. 331 p.

Reed, D. R., Bachmanov, A. A., & Tordoff, M. G. (2007). Forty mouse strain survey of body composition. *Physiology & behavior*, *91(5)*, 593–600.

Ricklefs, R. E. (2010). Embryo growth rates in birds and mammals. *Functional Ecology*, *24(3)*, 588–596.

Rohwäder, M.-S., & Jeltsch, F. (2022). Foraging personalities modify effects of habitat fragmentation on biodiversity. *Oikos*, e09056.

Saltelli, A., Annoni, P., Azzini, I., Campolongo, F., Ratto, M., & Tarantola, S. (2010). Variance based sensitivity analysis of model output. design and estimator for the total sensitivity index. *Computer physics communications*, *181(2)*, 259–270.

Savage, V. M., Gillooly, J. F., Woodruff, W. H., West, G. B., Allen, A. P., Enquist, B. J., & Brown, J. H. (2004). The predominance of quarter-power scaling in biology. *Functional Ecology*, *18(2)*, 257–282.

Schirmer, A., Herde, A., Eccard, J. A., & Dammhahn, M. (2019). Individuals in space: Personality-dependent space use, movement and microhabitat use facilitate individual spatial niche specialization. *Oecologia*, *189*, 647–660.

Schlägel, U. E., Grimm, V., Blaum, N., Colangeli, P., Dammhahn, M., Eccard, J. A., Hausmann, S. L., Herde, A., Hofer, H., Joshi, J., Et al. (2020). Movement-mediated community assembly and coexistence. *Biological Reviews*, *95(4)*, 1073–1096.

Schmidt-Nielsen, K. (1997). *Animal physiology: Adaptation and environment*. Cambridge university press.

Schulte-Hostedde, A., Millar, J., & Hickling, G. (2001). Evaluating body condition in small mammals. *Canadian Journal of Zoology*, *79(6)*, 1021–1029.

Shingleton, A. (2010). Allometry: The study of biological scaling. *Nature Education Knowledge*, *3(10)*, 2.

Sibly, R. M., Grimm, V., Martin, B. T., Johnston, A. S., Kułakowska, K., Topping, C. J., Calow, P., Nabe-Nielsen, J., Thorbek, P., & DeAngelis, D. L. (2013). Representing the acquisition and use of energy by individuals in agent-based models of animal populations. *Methods in Ecology and Evolution*, *4(2)*, 151–161.

Sobol, I. (2000). On sensitivity estimation for nonlinear mathematical models. *Matematicheskoe Modelirovanie*, *2*, 112–118.

Speakman, J. R. (1999). The cost of living: Field metabolic rates of small mammals. *Advances in ecological research*, *30*, 177–297.

Speakman, J. R. (2008). The physiological costs of reproduction in small mammals. *Philosophical Transactions of the Royal Society B: Biological Sciences*, *363(1490)*, 375–398.

Sutherland, G. D., Harestad, A. S., Price, K., & Lertzman, K. P. (2000). Scaling of natal dispersal distances in terrestrial birds and mammals. *Conservation ecology*, *4(1)*.

Szangolies, L., Gallagher, C. A., & Jeltsch, F. (2024). Individual energetics scale up to community coexistence: Movement, metabolism and biodiversity dynamics in fragmented landscapes. *Journal of Animal Ecology*.

Szangolies, L., Rohwäder, M.-S., & Jeltsch, F. (2022). Single large and several small habitat patches: A community perspective on their importance for biodiversity. *Basic and Applied Ecology*, *65*, 16–27.

Taylor, C. R., & Heglund, N. C. (1982). Energetics and mechanics of terrestrial locomotion. *Annual Review of Physiology*, *44(1)*, 97–107.

Teckentrup, L., Grimm, V., Kramer-Schadt, S., & Jeltsch, F. (2018). Community consequences of foraging under fear. *Ecological Modelling*, *383*, 80–90.

Tucker, M. A., Ord, T. J., & Rogers, T. L. (2014). Evolutionary predictors of mammalian home range size: Body mass, diet and the environment. *Global Ecology and Biogeography*, *23(10)*, 1105–1114.

Whittaker, R. (1975). *Communities and ecosystems*. New York: Macmillan.

Zhang, X.-Y., & Wang, D.-H. (2006). Energy metabolism, thermogenesis and body mass regulation in brandt's voles (lasiopodomys brandtii) during cold acclimation and rewarming. *Hormones and Behavior*, *50(1)*, 61–69.
